# *PROM1*, *CXCL8*, *RUNX1*, *NAV1* and *TP73* genes as independent markers predictive of prognosis or response to treatment in two cohorts of high-grade serous ovarian cancer patients

**DOI:** 10.1371/journal.pone.0271539

**Published:** 2022-07-22

**Authors:** Agnieszka Dansonka-Mieszkowska, Laura Aleksandra Szafron, Magdalena Kulesza, Anna Stachurska, Pawel Leszczynski, Agnieszka Tomczyk-Szatkowska, Piotr Sobiczewski, Joanna Parada, Mariusz Kulinczak, Joanna Moes-Sosnowska, Barbara Pienkowska-Grela, Jolanta Kupryjanczyk, Magdalena Chechlinska, Lukasz Michal Szafron

**Affiliations:** 1 Laboratory of Genetic and Molecular Cancer Diagnostics, Maria Sklodowska-Curie National Research Institute of Oncology, Warsaw, Poland; 2 Department of Cancer Biology, Maria Sklodowska-Curie National Research Institute of Oncology, Warsaw, Poland; 3 Department of Cancer Pathomorphology, Maria Sklodowska-Curie National Research Institute of Oncology, Warsaw, Poland; 4 Department of Gynecological Oncology, Maria Sklodowska-Curie National Research Institute of Oncology, Warsaw, Poland; 5 Cytogenetics Laboratory, Maria Sklodowska-Curie National Research Institute of Oncology, Warsaw, Poland; Tanta University Faculty of Medicine, EGYPT

## Abstract

Considering the vast biological diversity and high mortality rate in high-grade ovarian cancers, identification of novel biomarkers, enabling precise diagnosis and effective, less aggravating treatment, is of paramount importance. Based on scientific literature data, we selected 80 cancer-related genes and evaluated their mRNA expression in 70 high-grade serous ovarian cancer (HGSOC) samples by Real-Time qPCR. The results were validated in an independent Northern American cohort of 85 HGSOC patients with publicly available NGS RNA-seq data. Detailed statistical analyses of our cohort with multivariate Cox and logistic regression models considering clinico-pathological data and different *TP53* mutation statuses, revealed an altered expression of 49 genes to affect the prognosis and/or treatment response. Next, these genes were investigated in the validation cohort, to confirm the clinical significance of their expression alterations, and to identify genetic variants with an expected high or moderate impact on their products. The expression changes of five genes, *PROM1*, *CXCL8*, *RUNX1*, *NAV1*, *TP73*, were found to predict prognosis or response to treatment in both cohorts, depending on the *TP53* mutation status. In addition, we revealed novel and confirmed known SNPs in these genes, and showed that SNPs in the *PROM1* gene correlated with its elevated expression.

## Introduction

Ovarian carcinoma (OvCa) is the leading cause of death from gynecological malignancies worldwide. The mortality in this disease is exceptionally high, because the early-stage disease is usually asymptomatic while the symptoms in the late-stages are often nonspecific and there are no efficient screening methods. Thus, the majority of patients are diagnosed at late stages, which are characterized by poor prognosis. Despite nearly 70% remission rate after the first line of treatment, the disease recurs in about 50% of the patients, mostly due to chemoresistance [1]. Therefore, there is an urgent need to improve screening and therapeutic methods, and to achieve this goal, identification of new prognostic, predictive and screening markers, as well as novel therapeutic targets is essential. Considering the vast biological diversity of high-grade ovarian cancers, resulting in many apparently discrepant results, as clearly demonstrated herein in the discussion section, the discovery of universal, cohort-independent biomarkers is of paramount importance. As we have shown in our previous research on the *CEBPA* [2] and *EMSY* [3] genes, both expression changes and genetic alterations may affect ovarian cancer prognosis and/or response to chemotherapy. The clinical impact of such changes can also be mutually dependent, as observed for example in a nonsense-mediated mRNA decay (NMD), a gene regulatory mechanism which results in low concentrations of mRNAs transcribed from alleles carrying nonsense mutations [4]. Thus, in order to fully unravel the entire landscape of molecular interactions related to carcinogenesis and influencing the treatment outcome, the multifaceted approach needs to be applied. In the present study, the expression of a set of 80 cancer-related genes, nominated based on the scientific literature data, was evaluated by Real-Time qPCR in surgical tumor samples obtained from a uniform experimental cohort of previously untreated, high-grade serous ovarian cancer (HGSOC) patients, who were subsequently treated with the taxane/platinum (TP) chemotherapy. Expression alterations were analyzed in the context of patient overall survival (OS) and disease-free survival (DFS), as well as tumor sensitivity to chemical treatment (PS) or the chance of a complete remission (CR). Next, we validated our results in an independent cohort of HGSOC patients for whom the NGS RNA-seq data have been deposited in the public European Nucleotide Archive database (id: PRJNA396544) [5]. Noteworthy, the p53 accumulation status was also taken into account, since we have previously proved that it is a prominent confounding factor in the biomarker discovery [6, 7]. Missense mutations in the *TP53* gene result in the p53 protein accumulation which creates a permissive environment for the activity of oncogenes. In the present study, we found five genes, the changed expression of which concordantly affected patient outcomes in both cohorts. Furthermore, by using bioinformatic and statistical tools, we identified novel and known sequence variants in *TP53* and in the other analyzed genes, and correlated them with expression changes. This comprehensive study, summarizes the results obtained with two different techniques, i.e., Real-Time qPCR and NGS RNA-seq, and reveals significant relationships between genetic alterations, aberrant expression of the studied genes, and the clinical outcomes in two independent cohorts of HGSOC patients.

## Results

### Gene expression and ontology analysis

A list of 80 genes nominated for a Real-Time qPCR-based evaluation of expression in the experimental cohort is shown in the S1 Table. Forty nine of these genes were found differentially expressed depending on prognosis (OS and DFS of the patients) and/or prediction of treatment response (CR and PS of the tumors, see Table 1 and S2 for details). The genes with altered expression were also annotated and subjected to gene ontology analysis using the Database for Annotation, Visualization and Integrated Discovery (DAVID, v6.85) [8], which enabled their clustering based either on the pathways they are involved in (according to the Kyoto Encyclopedia of Genes and Genomes, KEGG [9]) or on UniProt keywords assigned to the protein product of each gene in the UniProt Knowledgebase (UniProtKB [10]). The detailed clustering results presented in the S3 Table showed that the genes with significantly altered expression in the experimental group of HGSOCs were predominantly associated with the p53 signaling pathway (p = 1.44e-13), cell cycle regulation (p = 2.19e-08), and conjugation of ubiquitin-like modifier proteins, e.g., ubiquitin (p = 4.42e-06). Some of the genes were also involved in the transcriptional misregulation observed in various cancers (p = 8.32E-05), the Hippo signaling pathway (p = 0.0044) and apoptosis (p = 0.0080). Remarkably, the p53 signaling pathway was characterized by the highest gene set enrichment among all categories reported by DAVID. This was an important premise supporting our approach to analyze gene expression in the context of p53 protein accumulation which results from missense mutations in the *TP53* gene [11]. In fact, the regression analyses performed in the subsets with and without *TP53* mutations revealed regularities undetectable when the entire group of patients was analyzed as a whole. Furthermore, the expression of three genes (*CD44*, *MKI67*, *RUNX2*) exerted opposing impact on DFS in the subgroups with different *TP53* mutation statuses (Table 1). In order to corroborate the gene expression results, the same regularities were searched for in an independent set of 85 HGSOC samples collected by Ducie et al. [5]. The expression changes have been successfully validated for five genes, *PROM1*, *CXCL8*, *RUNX1*, *NAV1*, and *TP73* (Table 2). Noteworthy, four other genes nominated for the expression validation (i.e., *CDK2*, *SNRPD3*, *TP53INP1*, *ZBTB8A*) had a number of reads too low to properly assess their mRNA expression. For each of these genes, the number of normalized NGS RNA-seq reads equaled 0 in over 90% of samples, which made the validation impossible (S4 Table).

**Table 1:** The selected significant results of the multivariate Cox and logistic regression analyses obtained for the experimental cohort of HGSOCs. The results confirmed in the validation cohort are emboldened and underlined. For all the significant results, refer to the S2 Table.

	All samples					TP53 missense mutation: no					TP53 missense mutation: yes				
Analysis	HR/OR	95% CI	p-value	N	Ev. no.	HR/OR	95% CI	p-value	N	Ev. no.	HR/OR	95% CI	p-value	N	Ev. no.
**DFS ~ PROM1**	59.1797	[3.66–956.967]	0.0041	35	32	-	-	-	-	-	**>1000**	**[45.57->1000]**	**0.0006**	**22**	**19**
FIGO IIIA-IIIB vs IIB-IIC	19.0412	[1.335–271.549]	0.0298	35	32	-	-	-	-	-	**221.4353**	**[4.088->1000]**	**0.0080**	**22**	**19**
FIGO IIIC vs IIB-IIC	-	-	-	-	-	-	-	-	-	-	**108.8040**	**[2.608->1000]**	**0.0138**	**22**	**19**
FIGO IV vs IIB-IIC	28.6215	[1.711–478.705]	0.0196	35	32	-	-	-	-	-	**>1000**	**[12.00->1000]**	**0.0033**	**22**	**19**
RT <2 cm vs 0 cm	2.7335	[1.001–7.462]	0.0497	35	32	-	-	-	-	-	-	-	-	-	-
**OS ~ PROM1**	-	-	-	-	-	9.2416	[1.274–67.005]	0.0278	21	21	-	-	-	-	-
**OS ~ CXCL8**	-	-	-	-	-	**34.5639**	**[1.560–765.542]**	**0.0250**	**21**	**21**	-	-	-	-	-
**DFS ~ RUNX1**	-	-	-	-	-	**<0.001**	**[<0.001–0.395]**	**0.0257**	**13**	**13**	-	-	-	-	-
**CR ~ NAV1**	**0.1461**	**[0.036–0.591]**	**0.0070**	**50**	**34**	-	-	-	-	-	<0.001	[0–0.462]	0.0278	29	21
**PS ~ NAV1**	**0.2614**	**[0.08–0.851]**	**0.0259**	**50**	**29**	-	-	-	-	-	<0.001	[0–0.524]	0.0310	29	19
**OS ~ NAV1**	-	-	-	-	-	-	-	-	-	-	>1000	[1.971->1000]	0.0371	29	23
RT <2 cm vs 0 cm	-	-	-	-	-	-	-	-	-	-	15.7298	[1.814–136.36]	0.0124	29	23
RT >2 cm vs 0 cm	-	-	-	-	-	-	-	-	-	-	28.4444	[2.868–282.08]	0.0042	29	23
**PS ~ TP73**	**0.5878**	**[0.347–0.996]**	**0.0482**	**69**	**45**	-	-	-	-	-	-	-	-	-	-
RT >2 cm vs 0 cm	**0.0861**	**[0.011–0.66]**	**0.0183**	**69**	**45**	-	-	-	-	-	-	-	-	-	-
**DFS ~ CD44**	20.9970	[1.194–369.375]	0.0374	35	32	<0.001	[<0.001–0.261]	0.0385	13	13	283.0698	[5.55->1000]	0.0049	22	19
FIGO IV vs IIB-IIC	-	-	-	-	-	-	-	-	-	-	34.5188	[1.112->1000]	0.0433	22	19
RT <2 cm vs 0 cm	3.0909	[1.089–8.77]	0.0339	35	32	-	-	-	-	-	5.1171	[1.031–25.407]	0.0458	22	19
**DFS ~ MKI67**	-	-	-	-	-	<0.001	[<0.001–0.185]	0.0239	13	13	484.7565	[4.687->1000]	0.0090	22	19
RT <2 cm vs 0 cm	-	-	-	-	-	-	-	-	-	-	6.3294	[1.188–33.731]	0.0307	22	19
**OS ~ MKI67**	-	-	-	-	-	<0.001	[<0.001–0.008]	0.0022	21	21	-	-	-	-	-
FIGO IIIA-IIIB vs IIB-IIC	-	-	-	-	-	13.4236	[1.334–135.012]	0.0274	21	21	-	-	-	-	-
**DFS ~ RUNX2**	-	-	-	-	-	<0.001	[<0.001–0.288]	0.0313	13	13	>1000	[38.66->1000]	0.0159	22	19
FIGO IIIA-IIIB vs IIB-IIC	-	-	-	-	-	-	-	-	-	-	569.4710	[3.926->1000]	0.0125	22	19
FIGO IIIC vs IIB-IIC	-	-	-	-	-	-	-	-	-	-	199.4673	[2.43->1000]	0.0185	22	19
FIGO IV vs IIB-IIC	-	-	-	-	-	-	-	-	-	-	>1000	[11.118->1000]	0.0036	22	19

Abbreviations used: CR–Complete Remission; DFS–Disease-Free Survival, FIGO–clinical stage; OS–Overall Survival; PS–Platinum Sensitivity; RT–Residual Tumor; HGSOC–high-grade serous ovarian cancer; HR–Hazard Ratio; OR–Odds Ratio; CI–Confidence Interval; Ev. no.–number of events (deaths, recurrences, PSs, CRs)

**Table 2:** Concordant DESeq2 results of the gene expression analysis in the validation HGSOC cohort.

Symbol	Ensembl.ID	Entrez	Analysis	Subset	baseMean	Log2FC	lfcSE	stat	pvalue	FC
**PROM1**	ENSG00000007062	8842	Recurrence: Yes vs No	*TP53* missense mut = = Yes	1200.3468	1.7814	0.4312	4.1311	0.00004	3.4376
**CXCL8**	ENSG00000169429	3576	Death: Yes vs No	*TP53* missense mut = = No	913.6175	1.9430	0.8468	2.2945	0.02176	3.8450
**RUNX1**	ENSG00000159216	861	Recurrence: Yes vs No	*TP53* missense mut = = No	8957.3244	-0.6968	0.3266	-2.1336	0.03287	0.6169
**NAV1**	ENSG00000134369	89796	PS: Yes vs No	All samples	504.9143	-0.6922	0.2357	-2.9363	0.00332	0.6189
**TP73**	ENSG00000078900	7161	PS: Yes vs No	All samples	360.8102	-0.8090	0.3638	-2.2236	0.02618	0.5708

Abbreviations used: PS–Platinum Sensitivity; FC–Fold Change; HGSOC–high-grade serous ovarian cancer

**Fig 1:**
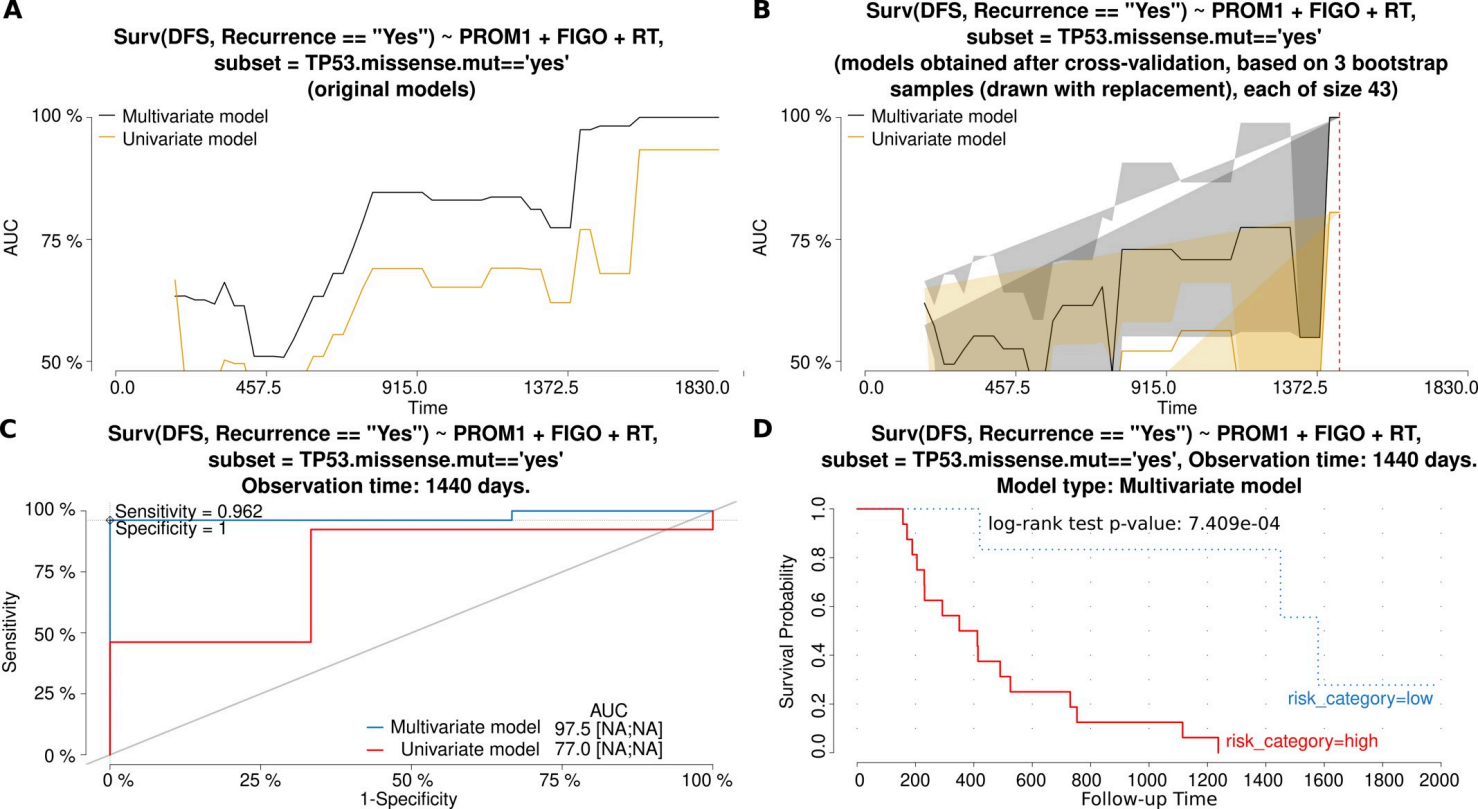
Characteristics and comparison of the Cox regression models (gene: *PROM1*). The models allowed for the assessment of the risk of **recurrence**, depending on either a single independent variable (*PROM1* mRNA expression (exp)–the univariate model or three independent variables (exp, FIGO and RT)–the multivariate model. Fig A and B show how the AUC values (and thus also discriminating abilities of each model) change in time for original models (A) and models obtained after a bootstrap-based cross-validation of the original data set (B). The bigger the AUC, the higher the performance of a model. A red dashed line marks the same time point which was used to draw the time-dependent ROC curve (C) for both models. In Fig C, an optimal cut-off point was calculated for the multivariate model based on the Youden index. Sensitivity and specificity for this cut-off point are also provided. In addition, AUC values [%] are listed alongside the 95% CI values, shown in square brackets, if calculable. Fig D depicts the Kaplan-Meier survival curves obtained for the multivariate model at the same time point as in the remaining plots. The risk was classified as either higher (high) or lower (low) than in the cut-off point. The Kaplan-Meier curves are supplemented with the result of the log-rank test, as well. Abbreviations used: DFS–Disease-Free Survival, FIGO–clinical stage; RT–Residual Tumor.

Overexpression of the first validated gene, *PROM1*, was shown to increase the risk of recurrence of tumors with the *TP53* missense mutations. This correlation was independent of a high FIGO stage, another factor of poor prognosis in this multivariate Cox regression model (Table 1). In Fig 1, the characteristics and comparison of the univariate and multivariate models is presented, with AUC plots for the original and bootstrap cross-validated models, and ROC and Kaplan-Meier curves. Additionally, the elevated expression of *PROM1* was a negative prognostic factor in the entire experimental cohort, and in the subgroup without missense mutations in *TP53*, though these results were not confirmed in the validation cohort. Interestingly, the *PROM1* expression did not affect a tumor response to the TP therapy.

**Fig 2:**
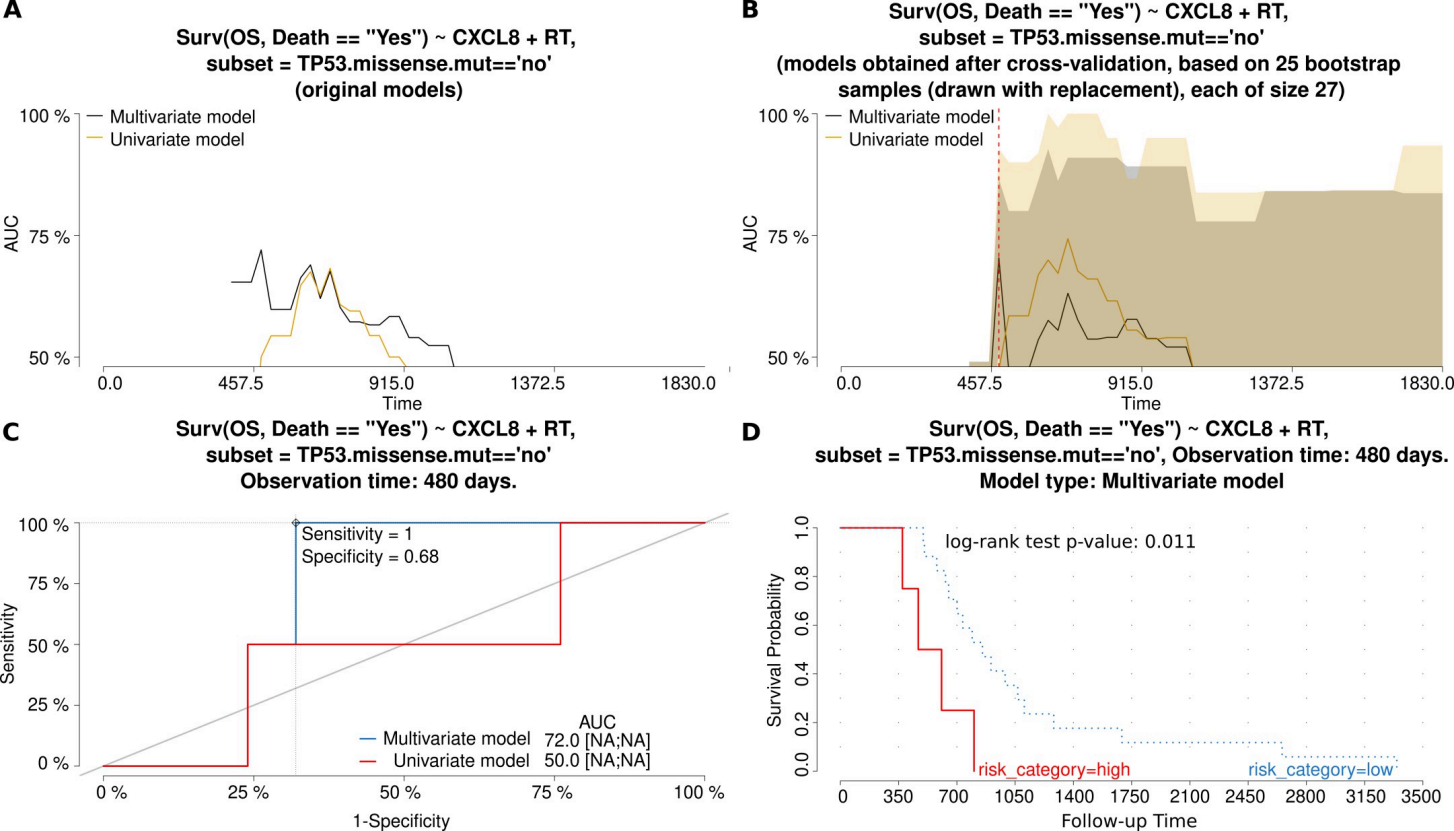
Characteristics and comparison of the Cox regression models (gene: *CXCL8*). The models allowed for the assessment of the risk of **death**, depending on either a single independent variable (*CXCL8* mRNA expression (exp)–the univariate model or three independent variables (exp, FIGO and RT)–the multivariate model. Fig A and B show how the AUC values (and thus also discriminating abilities of each model) change in time for original models (A) and models obtained after a bootstrap-based cross-validation of the original data set (B). The bigger the AUC, the higher the performance of a model. A red dashed line marks the same time point which was used to draw the time-dependent ROC curve (C) for both models. In Fig C, an optimal cut-off point was calculated for the multivariate model based on the Youden index. Sensitivity and specificity for this cut-off point are also provided. In addition, AUC values [%] are listed alongside the 95% CI values, shown in square brackets, if calculable. Fig D depicts the Kaplan-Meier survival curves obtained for the multivariate model at the same time point as in the remaining plots. The risk was classified as either higher (high) or lower (low) than in the cut-off point. The Kaplan-Meier curves are supplemented with the result of the log-rank test, as well. Abbreviations used: OS–Overall Survival; RT–Residual Tumor.

**Fig 3:**
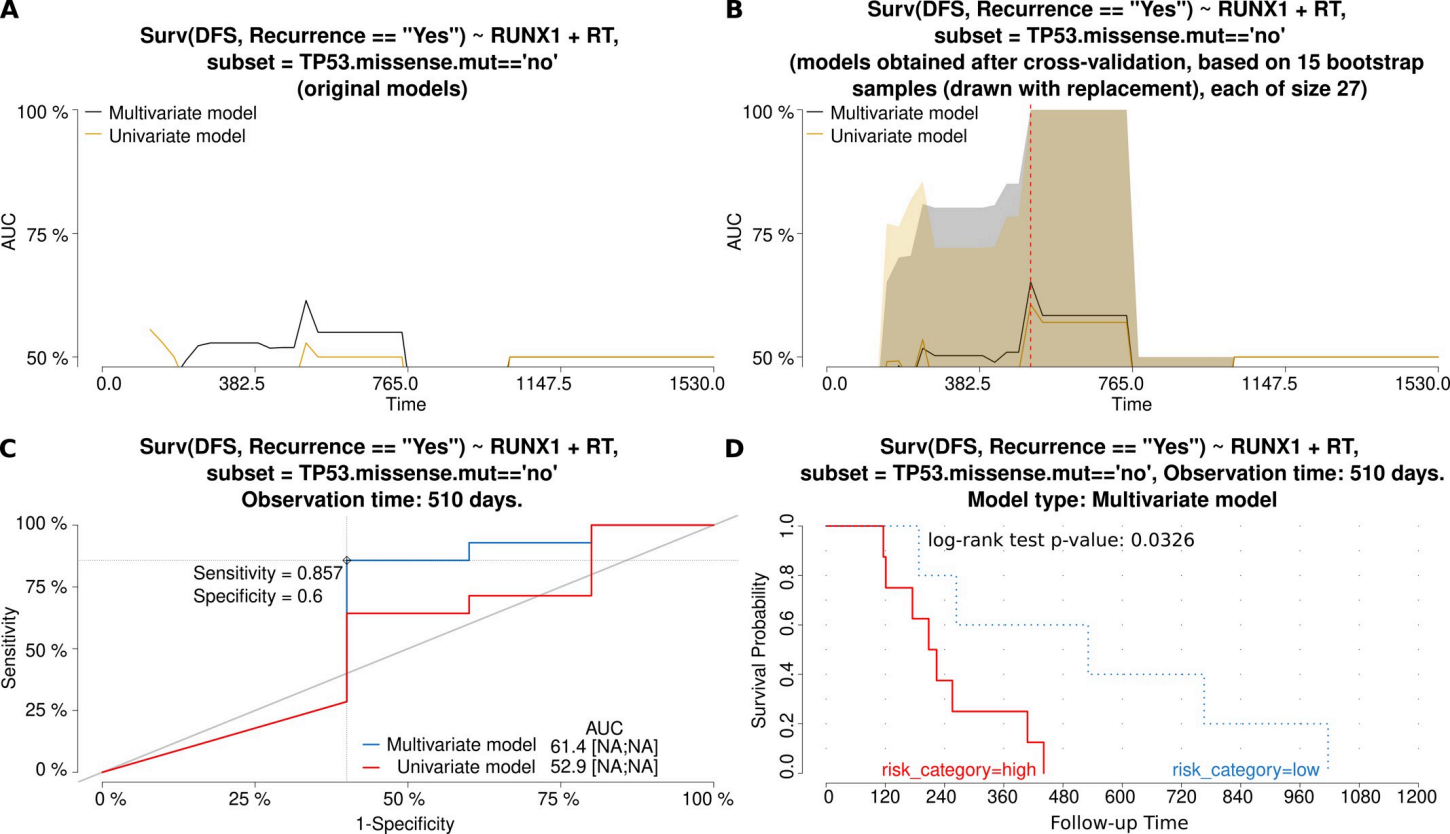
Characteristics and comparison of the Cox regression models (gene: *RUNX1*). The models allowed for the assessment of the risk of tumor **recurrence**, depending on either a single independent variable (*RUNX1* mRNA expression (exp)–the univariate model or three independent variables (exp, FIGO and RT)–the multivariate model. Fig A and B show how the AUC values (and thus also discriminating abilities of each model) change in time for original models (A) and models obtained after a bootstrap-based cross-validation of the original data set (B). The bigger the AUC, the higher the performance of a model. A red dashed line marks the same time point which was used to draw the time-dependent ROC curve (C) for both models. In Fig C, an optimal cut-off point was calculated for the multivariate model based on the Youden index. Sensitivity and specificity for this cut-off point are also provided. In addition, AUC values [%] are listed alongside the 95% CI values, shown in square brackets, if calculable. Fig D depicts the Kaplan-Meier survival curves obtained for the multivariate model at the same time point as in the remaining plots. The risk was classified as either higher (high) or lower (low) than in the cut-off point. The Kaplan-Meier curves are supplemented with the result of the log-rank test, as well. Abbreviations used: DFS–Disease-Free Survival, RT–Residual Tumor.

The elevated expression of *CXCL8* was associated with the increased risk of death, but only in patients with tumors lacking *TP53* missense alterations. By contrast, overexpression of *RUNX1* diminished the risk of recurrence in the same group of patients. Neither of these two genes was proved to influence ovarian cancer treatment response. For both genes, the characteristics and comparison of the univariate and multivariate models is presented in Figs 2 and 3, respectively, with AUC plots for the original and bootstrap cross-validated models, and ROC and Kaplan-Meier curves.

**Fig 4:**
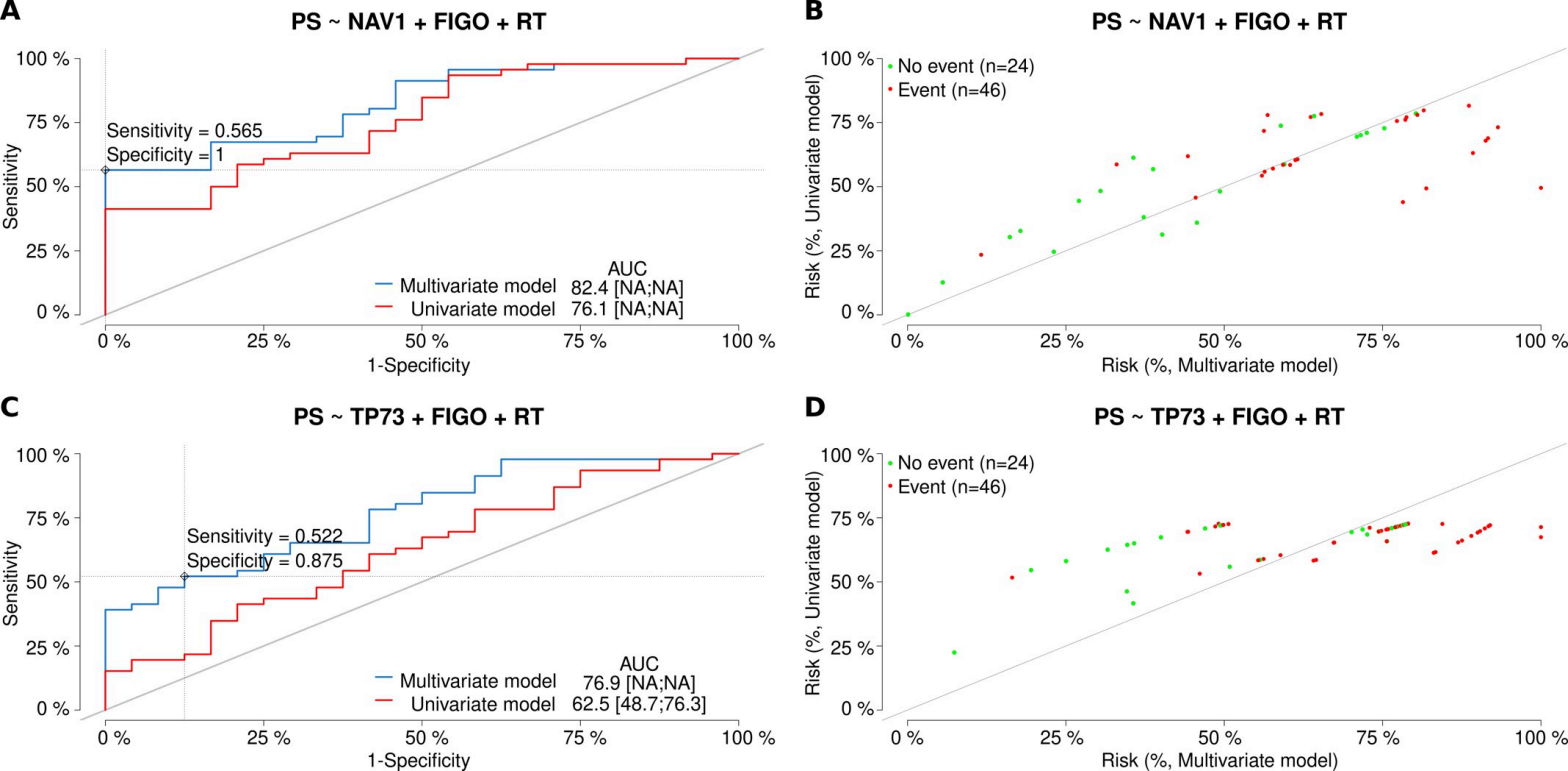
Characteristics and comparison of the logistic regression models (genes: *NAV1*, *TP73*). The models allowed for the assessment of **platinum sensitivity (PS)**, depending on either a single independent variable (mRNA expression (exp)–the univariate model or three independent variables (exp, FIGO and RT)–the multivariate model. In Fig A, ROC curves for both models for the *NAV1* gene are presented. An optimal cut-off point was calculated for the multivariate model based on the Youden index. Sensitivity and specificity for this cut-off point are also provided. In addition, AUC values [%] are listed alongside the 95% CI values, shown in square brackets, if calculable. Fig B compares discriminating capabilities of both the univariate and multivariate models for the *NAV1* gene. Fig C and D depict the results of the same analyses as Fig A and B but for the *TP73* gene. Abbreviations used: PS–Platinum Sensitivity; FIGO–clinical stage; RT–Residual Tumor.

As to the potential factors predictive of treatment response, elevated expression of the *NAV1* and *TP73* genes was demonstrated to decrease the chance of PS in the entire experimental cohort. The characteristics and comparison of the univariate and multivariate models for both genes is shown in Fig 4. Performances of the multivariate models before and after a bootstrap-based cross-validation were also comparable, equaling 82.4% and 72.4% for *NAV1* and 76.9% and 65.6% for *TP73*, respectively. In addition, the TP73-including model revealed Residual Tumor (RT) >2 cm as an independent factor worsening drug response, while overexpression of the *NAV1* gene was associated with the lower chance of CR in the same group of patients. Consistently, *NAV1* overexpression also increased the risk of death in the subgroup with missense mutations in *TP53*, though this regularity was not confirmed in the validation cohort of HGSOCs.

### Time trend analysis

No time trends were found for the frequencies of death, recurrence, complete remission or sensitivity to chemotherapy in the experimental cohort (S1 Fig). Information on time trends for the validation cohort was not provided in the study by Ducie et al. [5]. Unfortunately, we were unable to evaluate time trends for this cohort ourselves, since the dates of surgical cytoreduction of tumor masses for the patients in the validation cohort were also missing in the aforementioned study.

### Genetic variant effect predictions with Ensembl VEP

All the genes, the altered expression of which correlated with either cancer prognosis or treatment response prediction in the experimental cohort, were also subjected to the analysis of genetic variants with the Ensembl VEP app in the validation cohort. In summary, 716 high or moderate SNP changes were identified in 31 of 49 genes analyzed, 186 of which were of a high impact. Twenty eight of all these SNPs were unique, novel variants, previously not reported in the Ensembl database. Considering non-SNP alterations in the same cohort and gene set, six genetic changes were found in only two genes, *MUC16* and *TP53*, all of a high impact. Two unique, previously unreported variants were identified in this subset, both in the *TP53* gene (chr17:g.7676061dup and chr17:g.7675205del). As to the five genes characterized by concordant expression profiles in both cohorts, *PROM1*, *CXCL8*, *RUNX1*, *NAV1*, and *TP73*, in total, they harbored 24 SNP changes (including 4 unique, novel variants, i.e., chr4:g.15980539T>A (gene: *PROM1*, impact: high); chr1:g.201780560G>C (gene: *NAV1*, impact: high); chr1:g.201809166C>A (gene: *NAV1*, impact: moderate); chr1:g.201793831A>G, (gene: *NAV1*, impact: moderate)), and 0 non-SNP alterations. The detailed results of the VEP analysis are presented on heatmaps (S2 Fig), in Table 3, and in S4 and S5 Tables. We revealed no significant changes in gene alteration frequencies in the context of patient death or tumor recurrence and PS for any of the five genes with concordant expression profiles in both examined cohorts. Interestingly, by using the same method of statistical inference, we discovered that the tumors from the experimental cohort were more frequently mutated in the *TP53* locus than those from the validation cohort, and that the experimental cohort was also characterized by a less favorable outcome (a higher risk of patient death). The Pearson’s Chi-squared test with Yates’ continuity correction p-values equaled 0.0006 and 5.361e-15, respectively.

**Table 3:** Summary of the VEP analysis in the validation cohort for 5 genes with confirmed expression alterations and for *TP53*. The results for the other 43 genes analyzed are shown in S4 Table.

Gene	Significant	Validated	SNP variants (high)	SNP variants (high/ mod)	New unique SNP variants (high)	New unique SNP variants (high/ mod)	NON-SNP variants (high)	NON-SNP variants (high/ mod)	New unique NON-SNP variants (high)	New unique NON-SNP variants (high/ mod)	Altered samples (high)	Freq. of altered samples (high)	Altered samples (high/ mod)	Freq. of altered samples (high/ mod)
**PROM1**	Yes	Yes	9	10	1	1	0	0	0	0	9	0.106	10	0.118
**CXCL8**	Yes	Yes	2	2	0	0	0	0	0	0	2	0.024	2	0.024
**RUNX1**	Yes	Yes	1	2	0	0	0	0	0	0	1	0.012	2	0.024
**NAV1**	Yes	Yes	1	9	1	3	0	0	0	0	1	0.012	8	0.094
**TP73**	Yes	Yes	0	1	0	0	0	0	0	0	0	0.000	1	0.012
**TP53**	Yes	No	6	54	0	0	5	5	2	2	11	0.129	59	0.694

Abbreviations used: VEP–Variant Effect Prediction

### Relationships between genetic changes and expression alterations

**Fig 5:**
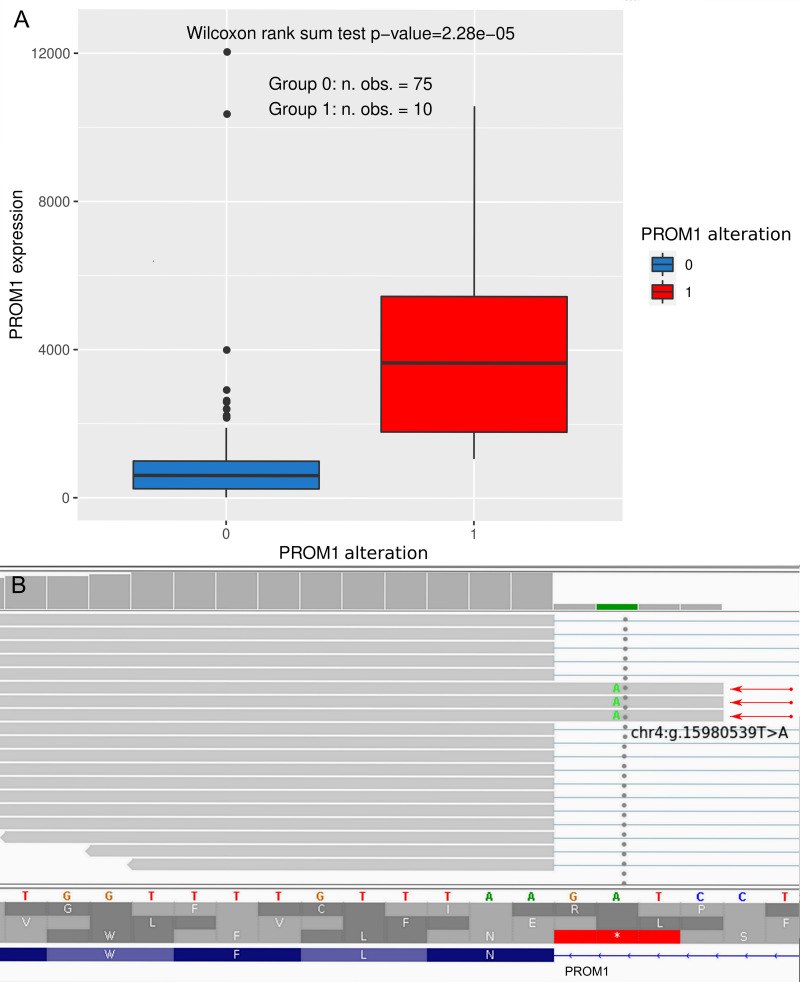
*PROM1* gene analysis. The *PROM1* gene alterations (denoted by “1”) and overexpression were found to be positively correlated (A). In Fig B, the chr4:g.15980539T>A (ENST00000447510.7:c.2374-2A>T) genetic alteration is shown, being a novel, splice acceptor variant, leading to the formation of an abnormal mRNA transcript (marked with red arrows). Since the *PROM1* gene is encoded by the minus DNA strand, the reference sequence of this strand is displayed.

Finally, we looked for the relationships between the presence of genetic changes in each of the five aforementioned genes and alterations in their expression. The significant results were obtained for the *PROM1* gene only, revealing a positive correlation between the occurrence of genetic variants and mRNA overexpression (Fig 5A). Noteworthy, nine of ten samples with the *PROM1* alterations harbored the same, previously undescribed SNP, classified by Ensembl as a splice acceptor variant with the high expected impact on the protein sequence (chr4:g.15980539T>A, ENST00000447510.7:c.2374-2A>T). Remarkably, this genetic change leads, in fact, to alternative splicing of the *PROM1* gene, resulting in the formation of an abnormal transcript, detectable in the NGS RNA-seq analysis (Fig 5B).

## Discussion

Based on the data available in the scientific literature, 80 genes were nominated for evaluation of their mRNA expression in pre-treatment tumors from an experimental cohort of the TP-treated HGSOC patients. This step involved the Real-Time qPCR analysis and detailed statistical inference with multivariate Cox and logistic regression models in subgroups with different *TP53* mutation statuses. The analysis revealed 49 genes the altered expression of which affected prognosis and predicted treatment response. Next, the same genes were investigated in an independent cohort of HGSOCs for alterations of their mRNA expression and identification of known and novel genetic variants with an expected high or moderate impact on their protein products’ sequences and functions. This led to identification of five genes, *PROM1*, *CXCL8*, *RUNX1*, *NAV1*, and *TP73*, with concordant expression profiles in both the experimental and validation cohorts. In these genes, we not only found 24 SNP variants in total (including four unique, novel changes) but also proved that SNPs in the *PROM1* gene correlated with its elevated expression which, in turn, was a negative prognostic factor in patients with HGSOCs harboring missense mutations in *TP53*.

The first question that needs to be discussed is the relatively low fraction of genes the altered expression of which was successfully confirmed in the validation cohort (5/49 = 10.2%). This might relate to several factors. First, the experimental and validation cohorts came from completely distinct populations (European vs Northern American). The so-called genetic background, being specific for each human population, is a well-known factor affecting susceptibility to ovarian cancer and other neoplasms [12, 13], as well as influencing the clinical outcome, including the risk of death or recurrence [14]. Another issue is the amount of stromal cell contamination (scc) in both cohorts. In the paper by Ducie et al. [5], the authors stated that all samples in the validation cohort had at least 60% of cancer cells, while in our (experimental) cohort all specimens had over 85% of cancer cells, and the majority of them had almost no scc thanks to macrodissection. A higher content of scc in the validation cohort might negatively affect reliability of the gene expression analysis, especially for the genes highly over-expressed or downregulated in cancer cells compared to stromal cells. Furthermore, despite both the mean and median NGS sequencing coverage in the validation cohort equaled about 58 million read pairs per sample (which is generally acknowledged as the high coverage for gene expression evaluation [15]), in four out of 49 analyzed genes, the number of reads was too low to properly assess their mRNA expression. This outcome suggests that for some genes, especially those of low expression, it would be better to further increase the NGS sequencing coverage, as also proposed by Kukurba and Montgomery [16]. Finally, the relatively low number of validated genes is likely due to some subtle differences between both cohorts in patient treatment, including surgical intervention, adjuvant chemotherapy, and collection and categorization of clinico-pathological data. For example, in the validation cohort, there were no data on CR. In addition, 15 tumors lacked the information on their treatment sensitivity, while in 11 other samples the FIGO stage was defined as “III”. In the experimental cohort, all these data were available for each specimen, and the tumors with FIGO IIIC (as more aggressive) were analyzed separately from those characterized by FIGO IIIA-B. Another important difference between the two cohorts involved tumor recurrence which was assessed for all the patients in the validation cohort. This seems to suggest that all patients in the Northern American cohort achieved a complete remission, while in the Polish (experimental) cohort 19 of 70 tumors (27.1%) did not respond positively to the TP therapy. Accordingly, the OS rate of the patients from the validation cohort (77.4%) was also significantly higher than in the experimental group (12.9%), despite the similar median OS time values. This unequivocally better response to treatment and prognosis observed in the Northern American cohort of HGSOCs is in line with the lower ovarian cancer mortality rate in the USA than in Poland [17], which may relate to diagnostic and therapeutic methods used. In step with this assumption, the frequency of cases with no residual disease in the experimental cohort (21.4%) was significantly lower than in the validation cohort (83.5%).

There is, however, another interesting variation between the two cohorts investigated herein, the prevalence of the *TP53* mutations. The fraction of tumors harboring *TP53* mutations in the experimental and validation cohorts equaled 92.9% and 69.4%, respectively, and the difference was statistically significant. The lower frequency of the *TP53* mutations in the validation cohort may reflect the phenomenon known as a nonsense-mediated mRNA decay, leading to low concentrations of mRNAs transcribed from alleles carrying nonsense mutations [4]. Given the fact that the VEP analysis in the experimental and validation cohorts was performed on either DNA or RNA, respectively, the number of nonsense mutations in *TP53* and in the other genes in the latter group can be underestimated. p53 is called the guardian of the genome for its imperative role in cancer prevention. The normal p53 protein partakes in the regulation of proliferation, apoptosis and DNA repair. It is also associated with the control of cell metabolism, autophagy and cell senescence [18]. On the other hand, when altered, p53 can acquire new capabilities (gain-of-function mutations) [19], and when it becomes nonfunctional, an environment promoting the activity of oncogenes is created, as shown in our previous research [6, 7].The occurrence of *TP53* mutations, especially of the missense type, considerably speeds up tumorigenesis [20]. In line with these findings, three oncogenes analyzed in the experimental cohort, i.e., *CD44*, *MKI67* and *RUNX2*, exerted opposed effects on patient DFS depending on the *TP53* mutation status. Interestingly, the three genes revealed their adverse prognostic value in tumors harboring the *TP53* missense mutations, thus supporting the aforementioned superior role of p53 in carcinogenesis. By contrast, the positive impact of *CD44*, *MKI67* and *RUNX2* overexpression on HGSOC patient survival is likely caused by their proliferation-promoting function which, in turn, sensitizes cancer cells with the normal p53 protein to cisplatin and other DNA-damaging agents [20]. In fact, the p53-mediated inhibition of CD44 was shown to enable untransformed cells to respond to stress-induced, p53-dependent cytostatic and apoptotic signals. By contrast, in transformed cells with impaired p53, CD44 became a key tumor-promoting agent [21]. Similarly, *MKI67* mRNA and Ki-67 protein expression were also found to be downregulated by the p53/p21 pathway upon DNA damage [22]. The expression of the mouse *Runx2* gene was demonstrated to be indirectly repressed by p53, leading to an inhibition of the osteogenic differentiation of bone marrow stromal cells. Consistently, the p53 loss sped up the differentiation process [23]. In summary, the expression of *CD44*, *MKI67* and *RUNX2* has previously been demonstrated to depend on the p53 protein function. This seems to provide a reasonable explanation of the apparent discrepancies in the prognostic value of these genes in subgroups with different *TP53* mutation statuses, found herein in the experimental HGSOC cohort. However, the prognostic meaning of all three oncogenes discussed in this paragraph was not confirmed in the validation cohort. The inconsistency could relate to the fact that, unlike the experimental group of HGSOC tumors, the validation series of samples comprised tumors obtained from patients cured in three different hospitals. This might negatively affect the uniformity of the validation cohort, and introduce some additional, hard-to-define variables, that ultimately could significantly interfere with the statistical results. For example, the differences in patient outcomes are likely to be influenced not only by the treating institution and the clinicians involved, but also by the date of patient admittance to the hospital. As stated above, the time trend analysis could not be performed for the validation cohort. Thereby, we were unable to determine whether and how the date of tumor excision affected the risk of patient death and tumor recurrence or the chance for a positive response to chemotherapy in this cohort. Considering the above, the findings related to the superior role of p53 in carcinogenesis, demonstrated herein for the *CD44*, *MKI67* and *RUNX2* genes, although supported by data available in the scientific literature, should be interpreted with caution.

The most prominent and interesting discovery of this study concerns five genes the altered expression of which was found to be associated with the clinical outcome, what was successfully validated in an independent cohort of HGSOC patients. The first of these genes, *TP73*, belongs to the evolutionarily oldest family of tumor suppressors, comprising also *TP53* and *TP63*. Considering over 70% similarity of the DNA-binding domains in p53, p63 and p73 proteins, many known p53 target genes (e.g., *CDKN1A* (*p21)*, *PUMA*, *NOXA*, *BAX* and *MDM2*) have been demonstrated to be regulated by the other members of this protein family, too. Nevertheless, the full repertoire of common and private target genes for each suppressor still needs to be unraveled [20]. Interestingly, the oligomerization domains in these proteins are much less conserved. As a result, p63 and p73 can form functional heterodimers, while their dimerization potential with p53 was proved to be limited [24]. Furthermore, in some p63 and p73 isoforms, an additional transactivation inhibitory domain (TID) was observed. This region, missing in the p53 protein, is considered to interact with the transactivation domain of the second protein partner of a dimer, thus inhibiting the transactivation properties of the entire complex [25]. Unlike the *TP53*, the *TP63* and *TP73* genes are rarely mutated in neoplastic cells [20]. Accordingly, in the validation cohort, we found one potentially harmful sequence variant in *TP73* (SNP with a moderate impact), no alterations in *TP63* (data not shown) and as much as 59 mutations in the *TP53* gene (Table 3 and S4). In contrast to the *TP53* gene, the mutations of which are linked to the Li-Fraumeni syndrome [26], *TP73* has not been associated with any hereditary disease so far, likely due to its involvement in many developmental and homeostatic processes, e.g., regulation of neural stem cell survival, self-renewal and differentiation in neurogenesis, regulation of multiciliogenesis, male and female reproduction, angiogenesis and immune response. *TP73* was also shown to prevent reactive oxygen species (ROS) accumulation [27]. The mechanism of p73 action in carcinogenesis still remains vague, as apparently contradictory functions have been assigned to this protein by different research groups, especially to its non-mutated, transcriptionally-active isoform (TAp73). On the one hand, this isoform is considered to suppress tumor angiogenesis by repressing proangiogenic and proinflammatory cytokines, as well as HIF1α [28]. On the other hand, the same isoform was discovered to positively regulate tumor angiogenesis, which might explain the surprisingly high prevalence of non-mutated, TAp73-overexpressing human tumors [29]. Our results support the latter study, portraying the *TP73* gene overexpression as a predictive marker of worse HGSOC response to the TP chemotherapy, independent of the *TP53* mutation status. The tumor-promoting role of *TP73* in ovarian cancer was also reported in two other studies [30, 31], and high expression of this gene was associated with advanced ovarian carcinoma when compared with the early-stage and borderline ovarian tumors. The involvement of *TP73* in chemotherapy response remains unclear, too. In some studies, TAp73 has been demonstrated to be an anti-apoptotic agent, inhibiting drug- and p53-induced apoptosis in ovarian and small-cell lung carcinomas [32, 33]. However, according to other reports, the TA-isoform of p73 plays a completely antithetical role as a crucial, positive mediator of platinum-induced apoptosis [34, 35]. The discrepancies as to the physiological function of TAp73 prove that further studies on this interesting and still poorly-investigated protein are necessary, not only to unravel the exact clinical meaning of p73 in carcinogenesis and chemotherapy resistance but also to fully comprehend subtle interactions between the members of the ancient p53-family of proteins.

*NAV1* (neuron navigator 1) was another gene found in our research to adversely affect HGSOC treatment response when overexpressed. Similarly to *TP73*, this impact was also independent of the *TP53* mutation status. Importantly, *NAV1* must not be confused with Nav 1.1–1.9 sodium channels proteins, which are encoded by *SCN1A*-*SCN11A* genes. So far, no scientific reports on the *NAV1* role in ovarian cancer have emerged. As to other cancer types, the *NAV1* promoter hypomethylation was observed in hormone receptor positive (expressing the estrogen receptor, ER+ and/or progesterone receptor, PR+) breast cancers [36] which seems to be consistent with our results. Studies on other genes belonging to the same family (*NAV2* and *NAV3*) showed their opposed roles in colon cancer development [37, 38]. The *NAV1* gene seems to exert pleiotropic effects. In mice, it was shown to be involved in the directional neuron migration process [39]. In humans, it was linked to the susceptibility to calcific aortic valve stenosis [40], and diabetes mellitus [41]. Given scarce scientific data on *NAV1* associations with cancer, we referred to the Pan-Cancer Analysis of Whole Genomes (PCAWG) study, based on an international collaboration to identify common patterns of mutations in more than 2,600 whole cancer genomes from the International Cancer Genome Consortium [42]. This study revealed that *NAV1* expression was elevated in ovarian carcinomas (N = 110) compared to normal ovaries (N = 39) (medians of transcripts per million (TPM) equaled 7 and 4, respectively). Thus, this outcome seems to be concordant with the negative predictive value of *NAV1* overexpression demonstrated herein. The NAV1 protein’s association with microtubules, reported by Martínez-López et al. [39], provides another suggestion that the HGSOC response to taxane (a microtubule depolymerization-inhibiting agent) may, indeed, depend on the *NAV1* gene expression levels.

The next validated gene, *CXCL8*, encodes Interleukin-8 (IL-8), a well-known chemotactic factor, a key mediator protein associated with inflammation, where it plays a pivotal role in neutrophil recruitment and degranulation [43]. Despite its generally acknowledged immunological function, the role of IL-8 in ovarian cancer is still obscure. Studies on cell lines showed the paclitaxel-induced IL-8 expression to retard the growth of ovarian cancer cells [44]. By contrast, some more recent research demonstrated that IL-8 promoted epithelial to mesenchymal transition (EMT) by increasing the MMP-2, MMP-9 and EpCAM expression, and stimulated the anchorage-independent growth, proliferation, angiogenic potential, adhesion and invasion in ovarian cancer cells [45–47]. In addition, the IL-8 knockdown was also shown to increase the HGSOC cells’ sensitivity to cisplatin [48]. Concordantly, the present study points to the oncogenic function of *CXCL8* overexpression in cells with no *TP53* missense mutations by showing its negative impact on overall survival of HGSOC patients. Other studies involving clinical material also corroborated our findings, by presenting a negative correlation between the survival time of ovarian cancer patients and the expression levels of IL-8 and IL-10 [49]. Moreover, IL-8 and its receptors (CXCR1 and CXCR2) are upregulated in advanced serous ovarian cancers [50].

The *RUNX1* gene, found herein to present significantly altered expression in both the experimental and validation cohorts, is involved in the generation of hematopoietic stem cells [51]. It has also been extensively investigated as a tumor suppressor in hematological cancers, where its locus is known for numerous chromosomal translocations [52]. However, genetic alterations in *RUNX1* also occur in many solid tumors. In gastric [53], hepatocellular [54], and esophageal [55] carcinomas, its tumor suppressor functions were revealed. Nevertheless, the role of *RUNX1* in breast cancer appears to be more ambiguous and hormone-dependent. On the one hand, tumor suppressor capabilities of *RUNX1* have been observed at the mRNA and protein levels in ER+ and/or PR+ breast cancers [56–58]. On the other hand, an increasing amount of evidence points to a pro-oncogenic role played by the *RUNX1* gene in breast cancer, intriguingly associated with the ER negative and triple negative (TN, i.e., ER-, PR-, HER2-) subtypes [59]. Accordingly, transcriptome studies have reported *RUNX1* mRNA upregulation in the TN breast carcinomas [60, 61]. The expression of the RUNX1 protein also increased with disease progression both in the TN tumor samples [62], and in a mouse model of breast cancer [63]. In ovarian cancer, *in vitro* studies showed that *RUNX1* inhibition in cell lines promoted cisplatin-induced apoptosis [64]. Moreover, the *RUNX1* knockdown significantly attenuated proliferative, migratory and invasive abilities of SKOV3 cells [65]. In line with these findings, hypomethylation of the *RUNX1* promoter was observed in HGSOC patients with chemoresistant tumors, suggesting its adverse, oncogenic role in cancer progression [66]. In addition, immunohistochemical analyses revealed that in tumors with high and low malignant potential, as well as in omental metastases, RUNX1 expression was significantly elevated, in contrast to normal ovaries [67]. Interestingly, our results in both, the experimental and validation cohorts suggest a tumor-suppressive role of *RUNX1* in HGSOCs, but only in the subgroup without missense mutations in *TP53*, where its elevated expression correlated with the decreased risk of tumor recurrence. The reasons for these seemingly antithetical outcomes can relate to hormone-dependence of ovarian neoplasms, just like in breast cancers. In line with this assumption, *RUNX1* alterations reported in the cBioPortal database (www.cbioportal.org) for ovarian cancers comprised both amplifications and deletions, thus highlighting the ambiguous and context-dependent role of the gene in this neoplasm [59]. One of the two genetic *RUNX1* changes identified herein in the VEP analysis of the HGSOC validation cohort was classified as a high-impact SNP (rs200431130), resulting in the loss of the *RUNX1* start codon in the recently predicted *RUNX1* transcript (XM_017028487.1). This discovery provides another supporting evidence for RUNX1 to conceivably play a tumor suppressor role in ovarian cancers.

The last of the validated genes, *PROM1*, encodes Prominin-1 (also known as CD133). CD133 is a glycoprotein frequently expressed in cancer stem cells [68]. The expression differs between different types of cancers. In pro-B acute lymphoblastic leukemia, and brain, esophageal, liver, testis, ovarian, and gastric cancers, CD133 is overexpressed while in other cancer types, including kidney cancer, CD133 is down-regulated [69]. Transcription of the human *PROM1* gene is driven by five alternative promoters, three of which are located on CpG islands and are partially regulated by methylation. The occurrence of several promoter regions leads to alternative splicing, resulting in CD133 structural variants with potentially unique roles [68]. In ovarian cancer, CD133 is a marker of poor prognosis and worse response to treatment. Studies on cell lines, animal models as well as on a clinical material have shown, that elevated levels of CD133 correlated with shorter DFS and OS, advanced disease stage, ascites accumulation, increased platinum resistance and higher risk of metastasis to peritoneum and the central nervous system [70–72]. Additionally, the CD133 expression was shown to correlate with ovarian tumor aggressiveness–the highest expression of CD133 was observed in malignant epithelial ovarian tumors when compared to borderline (with moderate expression) and benign tumors (low expression), as well as with a histological type–serous ovarian carcinomas showed the highest immunohistochemical expression score of CD133 [73]. Our results are fully consistent with the oncogenic potential of Prominin-1 demonstrated by other research groups, revealing the negative impact of *PROM1* gene overexpression on patient DFS in the subgroup of tumors with missense *TP53* mutations, found in both the experimental and validation cohorts. We also discovered a positive correlation between the elevated expression of *PROM1* and its SNPs. It is worth to note that nine out of ten samples with genetic changes within the *PROM1* gene harbored the same high-impact alteration: chr4:g.15980539T>A (ENST00000447510.7:c.2374-2A>T), being a novel, splice acceptor variant, resulting in the formation of an abnormal *PROM1* mRNA transcript. Splice site mutations have been extensively studied in recent years in various human disorders, including cancers. Abnormalities in alternative splicing were linked to tumorigenesis, as they induced a plethora of physiological alterations in neoplastic cells, including the imbalance between proliferation and apoptosis, increased invasiveness, angiogenic and metastatic potential as well as an elevated rate of metabolism, all of which belong to the well-known cancer hallmarks [74]. Splice site SNPs may contribute to the above-mentioned physiological changes by affecting the activity of cryptic sites, leading to the altered frequency of exon inclusion/exclusion, and promoting the alternate exon usage. As a result, the amounts of particular splice forms in the cell may either increase or diminish, as demonstrated for, e.g., *EMID1* and *IL19*, respectively, by Mucaki et al. [75]. Such a mechanism may also explain the correlation between the occurrence of splice site mutations and overexpression, reported herein for the *PROM1* gene.

In summary, in two independent cohorts of HGSOC patients, we identified five genes, *PROM1*, *CXCL8*, *RUNX1*, *NAV1* and *TP73*, as potential biomarkers to predict prognosis or response to treatment. This was achieved by combining the results of Real-Time qPCR analyses performed in our Polish cohort and the NGS RNA-seq studies carried out by Ducie et al. [5] in the American cohort (validation). This approach let us find cohort-independent factors affecting the clinical course of ovarian cancer. Moreover, our study further confirms the importance of the *TP53* mutation status for ovarian cancer biology and biomarker discoveries.

## Conclusions

The altered expression of five genes, *PROM1*, *CXCL8*, *RUNX1*, *NAV1* and *TP73*, affects patient prognosis or predicts treatment response. Remarkably, the context of the *TP53* missense mutations in the tumors is crucial to unravel these associations, which confirms our previous results, pointing to p53 as an important confounding determinant in the biomarker discovery. In addition, hundreds of genetic alterations with the expected high or moderate impact on the encoded proteins’ sequence and function were identified, including thirty unique changes so far unknown. Noteworthy, some of these variants are likely to affect the expression of the altered genes, as shown herein for the *PROM1* oncogene. Our results add to a better understanding of ovarian cancer-driving mechanisms, thus provide the grounds for the development of novel, targeted, less aggravating and, hopefully, more effective methods of treatment.

## Materials and methods

### Ethics statement

The study was conducted according to the guidelines of the Declaration of Helsinki, and approved in writing by the Institutional Review Board of the Maria Sklodowska-Curie National Research Institute of Oncology (nos. 49/2003 and 39/2007). Informed consent in writing was obtained from all subjects involved in the study.

### Patients and tumors

The Real-Time qPCR analysis was performed in a uniform series of 70 high-grade, serous, ovarian cancer samples from previously untreated patients (herein, the corresponding cohort of patients is named “experimental”). All patients were treated at a single institution, the Maria Sklodowska-Curie National Research Institute of Oncology, Warsaw, Poland in the years 1995–2006 and received TP chemotherapy after surgery. Medical records of all the patients were critically reviewed by at least two clinicians. The material was carefully selected to meet the following criteria: no chemotherapy before staging laparotomy, adequate staging procedure according to the recommendations by the International Federation of Gynecologists and Obstetricians (FIGO) [76], tumor tissue from the first laparotomy available, poor tumor differentiation, availability of clinical data including residual tumor size and follow-up. All tumors in the experimental cohort were uniformly histopathologically reviewed, classified according to the criteria of the World Health Organization [77] and graded according to the standards given by Barber et al. [78] in a four-grade scale which, although outdated, is more detailed (only tumors with grades 3 and 4 were included in this study). We decided not to use the up-to-date three-grade scale, since our previous research showed differences between grade 3 and 4 tumors with respect to patient overall survival [79]. Additionally, a complete evaluation of p53 status was performed using the PAb1801 mouse monoclonal antibody (1:500, Sigma-Genosys, Cambridge, UK), as described previously [6]. Accumulation of the p53 protein results predominantly from missense *TP53* mutations. Other mutations do not cause the p53 protein accumulation, just like in case of the wild-type *TP53* gene [80]. In the experimental cohort, there was approximately 91% correlation between the *TP53* missense mutations and the p53 protein accumulation. Out of 70 specimens that we examined, 46 (65.7%) exhibited the p53 accumulation, while the *TP53* missense mutations were found in 43 tumors (61.4%). For the evaluation of clinical endpoints in the experimental group, CR was defined as disappearance of all clinical and biochemical symptoms of ovarian cancer assessed after completion of the first-line chemotherapy and confirmed four weeks later [81]. DFS was assessed only for the patients who achieved a CR. All surviving patients had at least a 6-month follow-up.

In order to validate the gene expression results in an independent cohort of 85 HGSOC samples, we have examined the publicly available NGS RNA-seq data set (id: PRJNA396544) from the European Nucleotide Archive (ENA), deposited there by Ducie et al. [5]. Noteworthy, the study by Ducie et al. was performed on the biggest number of high-grade serous ovarian cancer samples (286 in total, including 85 samples used in the NGS RNA-seq experiments) from among the data sets available in the Gene Expression Omnibus (a database administered by the National Center for Biotechnology Information (NCBI), USA). Moreover, all these samples came from patients who did not undergo a chemical treatment before tumor excision, and were treated postoperatively with taxanes. Thus, this cohort was very similar to our (experimental) cohort of patients. Furthermore, Ducie et al. provided a detailed clinico-pathological characteristics of patients and tumors used in their study which let us compare the prognostic and predictive value of gene expression changes in both cohorts to identify cohort-independent biomarkers. Moreover, in the study by Ducie et al., the median and mean values of the number of NGS read clusters for the entire validation cohort both exceeded 50 millions, while modes of Phred sequencing quality scores ranged from 37 to 39. Thus, these data were good enough with respect to both the transcriptome coverage and sequencing quality. Last but not least, Ducie et al. have published their research in a high-ranked journal (Nature Communications). This should guarantee the rigorousness and thoroughness of the peer-review process, thereby ensuring the reliability of the paper itself and of all supplementary materials.

Remarkably, the clinico-pathological data available for the validation cohort differ, with respect to some variables, from the data we gathered for the experimental cohort, e.g., in the experimental cohort treatment response prediction was assessed based on two parameters: CR and PS, whereas for the validation cohort the information on complete tumor remission was missing. To overcome this limitation, the PS and CR variables in the experimental group were both compared with the PS in the validation cohort. Additionally, in the experimental cohort, OS and DFS times were calculated for each patient. By contrast, in the validation cohort OS was available but the DFS variable was missing, and the authors provided Progression-Free-Survival (PFS) data instead. As DFS and PFS variables cannot be treated interchangeably, we decided to compare the DFS in the experimental cohort with the recurrence status in the validation group. As to the validation procedure, in either prognosis or treatment response prediction, the results were considered as confirmed when HR/OR values in the experimental group and Fold Change (FC) values in the validation cohort were both either above or below 1 for the same gene, the same dependent variable (OS, DFS, CR, PS) and the same *TP53* mutation status. A detailed clinico-pathological characteristics of the patients and tumors in both the experimental and validation cohorts is presented in Table 4.

**Table 4:** Clinico-pathological characteristics of patients and tumors.

	Experimental cohort (N = 70)		Validation cohort (N = 85)	
Variable	N	Freq.	N	Freq.
**Age: Min.**	29	-	42	-
**Age: Max.**	79	-	79	-
**Age: Mean**	53	-	59	-
**Age: Median**	54	-	59	-
**CR: No**	19	0.271	-	-
**CR: Yes**	51	0.729	-	-
**Death: No**	9	0.129	65	0.765
**Death: Yes**	61	0.871	19	0.224
**Death: NA’s**	-	-	1	0.012
**DFS (days): Min.**	0	-	-	-
**DFS (days): Max.**	1989	-	-	-
**DFS (days): Mean**	432	-	-	-
**DFS (days): Median**	260	-	-	-
**FIGO: IA-IIC**	2	0.029	4	0.048
**FIGO: IIIA-IIIB**	6	0.086	2	0.024
**FIGO: IIIC**	56	0.8	43	0.506
**FIGO: III**	-	-	11	0.129
**FIGO: IV**	6	0.086	25	0.294
**OS (days): Min.**	296	-	132	-
**OS (days): Max.**	5002	-	2601	-
**OS (days): Mean**	1404	-	1080	-
**OS (days): Median**	1080	-	1072	-
**OS (days): NA’s**	-	-	1	-
**PFS (days): Min.**	-	-	120	-
**PFS (days): Max.**	-	-	2238	-
**PFS (days): Mean**	-	-	730	-
**PFS (days): Median**	-	-	633	-
**PS: No**	24	0.343	11	0.129
**PS: Yes**	46	0.657	59	0.694
**PS: NA’s**	-	-	15	0.176
**Recurrence: No**	6	0.086	28	0.329
**Recurrence: Yes**	45	0.643	57	0.671
**Recurrence: NA’s**	19	0.271	-	-
**RT: 0 cm**	15	0.214	71	0.835
**RT: < 2 cm**	40	0.571	-	-
**RT: > = 2 cm**	14	0.2	-	-
**RT: Suboptimal**	-	-	12	0.141
**RT: NA’s**	1	0.014	2	0.024
**p53 protein accumulation: No**	24	0.343	-	-
**p53 protein accumulation: Yes**	46	0.657	-	-
**TP53 missense mutation: No**	27	0.386	36	0.424
**TP53 missense mutation: Yes**	43	0.614	49	0.576
**TP53 mutation: No**	5	0.071	26	0.306
**TP53 mutation: Yes (missense)**	43	0.614	49	0.576
**TP53 mutation: Yes (non-missense)**	22	0.314	10	0.118

Abbreviations used: CR–Complete Remission; DFS–Disease-Free Survival, FIGO–clinical stage; OS–Overall Survival; PFS–Progression-Free Survival; PS–Platinum Sensitivity; RT–Residual Tumor

### Real-Time qPCR-based studies of gene expression

RNA was isolated from frozen tumor sections with over 85% of cancer cells, using the RNeasy Plus Mini Kit (Qiagen, Hilden, Germany). RNA quantity was measured with NanoDrop spectrophotometer (Thermo Fisher Scientific, Waltham, MA, USA), and its quality was assessed on Agilent Bioanalyzer (Agilent Technologies, Santa Clara, CA, USA). RNA integrity numbers (RINs) of the samples ranged from 6.5 to 9.4.

Based on data available in the scientific literature, 80 genes were nominated for their expression verification by Real-Time qPCR. The majority of the genes was analyzed in the entire experimental series of 70 samples, but some genes were assessed in a subset of the experimental series (51 samples or 50 samples). For the list of genes, their descriptions and series sizes, see the S1 Table. All Real-Time qPCR experiments described here were run in triplicates on either the 7500 Fast or the 7900HT Real-Time PCR Systems (both manufactured by Thermo Fisher Scientific). Gene expression was evaluated with TaqMan assays (Thermo Fisher Scientific) (for the list of assays, see the S1 Table). The expression of each gene analyzed herein was normalized to the geometric mean of expression of three reference genes, *HPRT1* (hypoxanthine phosphoribosyltransferase 1), *PPIA* (peptidylprolyl isomerase A), and *GUSB* (glucuronidase beta)) [82]. These three genes were nominated experimentally to be the most stably expressed among 11 house-keeping genes available on the TaqMan Human Endogenous Control Plates (Thermo Fisher Scientific), as assessed in 8 randomly selected ovarian tumors. The stability was calculated with the qBase^PLUS^ app (Biogazelle NV, Zwijnaarde, Belgium), utilizing an improved version of the geNorm algorithm [82, 83]. In every reaction, TaqMan Universal PCR Master Mix II with uracil-N-glycosylase (Thermo Fisher Scientific) was used to reduce the risk of cross-contamination with the products of previous PCRs. Each Real-Time qPCR reaction was performed according to the manufacturer’s recommendations, using 10–12 ng of total RNA (earlier reverse transcribed to cDNA with the High-Capacity cDNA Reverse Transcription Kit (Thermo Fisher Scientific)). The expression of all the analyzed genes was calculated with the ΔΔCt method for relative quantification of gene expression [84]. The calibrator was always the sample with the highest expression level, thus it was different for each gene. This approach ensured that the calibrated expression values ranged from 0 to 1 for all the genes.

### NGS RNA-seq studies of gene expression

The quality of the FASTQ files acquired for 85 HGSOC samples from the validation cohort was analyzed with the Fastqc app (version: 0.11.9) followed by adapter clipping and removal of poor-quality fragments of reads with the Trimmomatic app (version: 0.39). NGS reads were then mapped to a reference sequence of the human genome (assembly: GRCh38 (hg38)) using the HISAT2 sequence aligner (version: 2.2.1). After mapping, the PCR and optical duplicates were removed with the MarkDuplicates app, a part of the Genome Analysis Toolkit (GATK, version: 4.1.7.0). The assessment of mapping quality was carried out using Samtools (version: 1.12) and Qualimap (version: 2.2.2-dev) apps, while Integrative Genomics Viewer (IGV, version: 2.10.0) served for visualization of the mapping results. The DESeq2 package for R (version: 1.26.0) was employed for identification of differentially expressed genes. Importantly, due to the relatively low depth of sequencing coverage in the acquired NGS RNA-Seq data (median and average values of mean coverage depths calculated with the Qualimap app for the entire cohort equaled 9.4 and 9.3, respectively), the DESeq2 analysis had to be performed on unfiltered data. Otherwise, about two thirds of the genes (including some being significant in our Real-Time qPCR studies) would be filtered out and thus excluded from further analyses.

### Ensembl Variant Effect Predictor (VEP) for identification of sequence variants

In the present study, the aforementioned NGS RNAseq data set generated for the validation cohort was used not only to find significant alterations in gene expression, but also to determine sequence variants. The Variant Call Format (VCF) files were generated (with the Allele Depth tag, AD) using the bcftools (version: 1.12) and the GRCh38 (hg38) assembly of the human genome. Next, the variants were subjected to a two-step filtering. First, the variants less frequent than 10% were filtered out based on the AD tag, using the VAF checker app (version: 1.0) (a program developed by our team, and available for download at GitHub: https://github.com/lukszafron/VAF.checker). Then, the vcf-annotate app from the VCFtools package (version: 0.1.16) was employed to filter out the variants that do not meet the following criteria: all filters with default values applied except for: MinAB = 2 (a minimum number of alternate bases of 2), Qual = 20 (a minimum sequence quality of 20), and MinMQ = 20 (a minimum mapping quality of 20). Subsequently, the obtained VCF files were divided with bcftools into two subsets (SNP, and non-SNP), containing snp variants vs all other sequence alterations, i.e., indels (insertions or deletions), mnps (multi-nucleotide polymorphisms), bnd (breakpoints), and others, respectively. Next, the variant identification and effect prediction analysis was carried out using the Ensembl Variant Effect Predictor (VEP) app (version: 104.0, released in May 2021) and the merged Ensembl and RefSeq databases [85]. The obtained tab-delimited CSV files (VEP output tables) were further analyzed consecutively with two R programs developed by LMS, vep.r (version: 1.1), and vep.comparison.r (version: 1.0), both available for download at https://github.com/lukszafron. Ensembl VEP divides sequence variants into four categories: high, moderate, low and modifier, based on their expected impact on the transcript and protein sequences. For details, please refer to the web page [86]. The two aforementioned R apps were utilized first to filter out all variants characterized by low or modifier impacts, and then to exclude all variants except those which either had a known adverse clinical significance or negatively affected the protein structure and function (as assessed by either the SIFT or PolyPhen algorithms). The new, previously unidentified sequence variants (with an empty "Existing_variation" field in the VEP output table), variants with empty “CLIN_SIG”, “SIFT” and “PolyPhen” fields, or those with the maximum allele frequency (MAX_AF) lower than 0.01 were also included in the final report generated by the vep.r app. The analyses were carried out independently for SNP and non-SNP variants. Subsequently, these results were combined together with binarization of sequence alterations for every gene (sequence variants with a high or moderate impact present (1) vs absent (0)). Afterwards, statistical analyses were carried out, followed by the data visualization to identify genes with the frequency of sequence alterations significantly different between the investigated subgroups (characterized by distinct death, recurrence and PS statuses). This final step of the analysis was performed with the vep.comparison.r script.

To assess the *TP53* mutation status in the experimental cohort, we prepared NGS DNA-seq libraries, comprising exonic regions of 41 oncogenes and tumor suppressors (including *TP53*) involved in the development of hereditary ovarian cancer (the Ion AmpliSeq Comprehensive Ovarian Cancer Research Panel, Thermo Fisher Scientific). In those libraries, the gene set enrichment was carried out using the sequence capture technology and the SeqCap EZ Prime Choice Probes, both offered by Roche (Basel, Switzerland). The obtained NGS data were subsequently analyzed using the methods described above to find genetic variants with a high or moderate expected impact on the encoded proteins’ sequence and function.

### Statistical analysis

All statistical analyses were carried in the R environment (version: 3.6.1). The survival analysis in the experimental group of patients was performed using the multivariate Cox proportional hazards models (the survival package for R, version: 3.2–11). All Cox models were also checked with respect to proportionality of hazards for each variable used. The prediction of treatment response in the experimental group of patients was carried by generating multivariate logistic regression models (R packages: stats (version: 3.6.1) and rms (version: 6.2–0)). In order to verify the discriminating capabilities of the Cox and logistic regression models, we performed their cross-validation in new data sets, generated from the original data by bootstrapping (with replacement) and subsequent comparison of areas under curves (AUCs) between the original and bootstrapped data sets, using the riskRegression package for R (version: 2020.12.8) [87]. All the analyses were performed not only in the entire group of tumors, but also in the subgroups with and without missense mutations in the *TP53* gene, and were adjusted for the FIGO stage and RT. Noteworthy, for all the analyzed genes, the expression was treated as a continuous variable to avoid arbitrary categorization of data, which could potentially lead to unreliable statistical results. In case of continuous variables, contrary to categorical variables, HRs/ORs cannot be treated as the ratio of the hazard/odds rates, corresponding to the conditions described by two sets of explanatory variables. For continuous variables, the same interpretation applies to a unit difference [88]. As mentioned above, a tumor exhibiting the highest expression of a gene was used as a calibrator for this gene. Thus, all the expression values ranged from 0 to 1. This approach allowed the approximate estimation of the risks in a similar way as for categorical variables. However, it has to be underlined that the real HR/OR will always be lower from what is shown in this paper, because only one tumor (calibrator) has the gene expression of 1, and none–equaling 0. Time trends of OS, DFS, CR and PS were evaluated with the Mann-Kendall homogeneity test, and supported with the autocorrelation function (ACF) plots. In the validation cohort of 85 HGSOCs, the correlation between the mutation status of each gene and its expression level was assessed with the Wilcoxon rank sum test, while the changes in gene mutations’ distribution in the subgroups with different clinical outcomes were analyzed with either the Chi-squared or Fisher’s exact test depending on the subgroups’ sizes. The same method of statistical inference was employed to compare the frequencies of either *TP53* mutations or patient deaths in the experimental and validation cohorts. All p-values shown herein were considered significant at the statistical significance level (alpha) of 0.05. Given the use of two independent HGSOC cohorts to study the impact of altered gene expression on ovarian cancer prognosis and prediction of treatment response, we decided not to apply the Benjamini-Hochberg correction controlling the false discovery rate. Otherwise, the rate of the statistical type II error (beta) would increase with concurrent, unintended decrease of the statistical power (1-beta) [89].

## Supporting information

S1 Fig The analysis of prognostic and predictive time trends in the experimental cohort of ovarian cancer patients. The patients underwent their first surgical treatment in the years 1995–2006. Time trends concerned overall survival (OS) (A,B); disease-free survival (DFS) (C,D); sensitivity to chemotherapy (PS) (E,F) and complete remission (CR) (G,H). The trends are shown as a trend line of death, relapse, PS and CR frequencies, respectively, supplemented with the results of the Mann-Kendall homogeneity test, and supported with autocorrelation function (ACF) plots.
(TIF)

S2 Fig VEP analysis heatmaps. The results of the VEP analysis in the validation cohort of HGSOCs for 49 genes with significantly changed mRNA expression in the experimental cohort (only the genes with at least one sequence alteration in at least one sample are included). Abbreviations used: VEP–Variant Effect Prediction; HGSOCs–high-grade serous ovarian cancers.
(TIF)

S1 Table Eighty genes analyzed by Real-Time qPCR in the experimental cohort with corresponding TaqMan assay IDs. (XLSX)

S2 Table The significant results of the multivariate Cox and logistic regression analyses obtained for the experimental cohort of HGSOCs. (PDF)

S3 Table DAVID KEGG Pathways&UP Keywords analysis for 49 genes with altered expression in the experimental cohort. (XLS)

S4 Table Summary of the VEP analysis in the validation cohort for 49 genes differentially expressed in the experimental cohort. (PDF)

S5 Table New and known SNP and non-SNP variants of high or moderate impact in the validation cohort, found in 49 genes with altered expression in the experimental cohort. (XLSX)
