# Supplementary figures and images for: PROM1, CXCL8, RUNX1, NAV1 and TP73 genes as independent markers predictive of prognosis or response to treatment in two cohorts of high-grade serous ovarian cancer patients
Source: PLoS One. 2022 Jul 22;17(7):e0271539. doi: 10.1371/journal.pone.0271539 (PMC9307210; doi:10.1371/journal.pone.0271539)

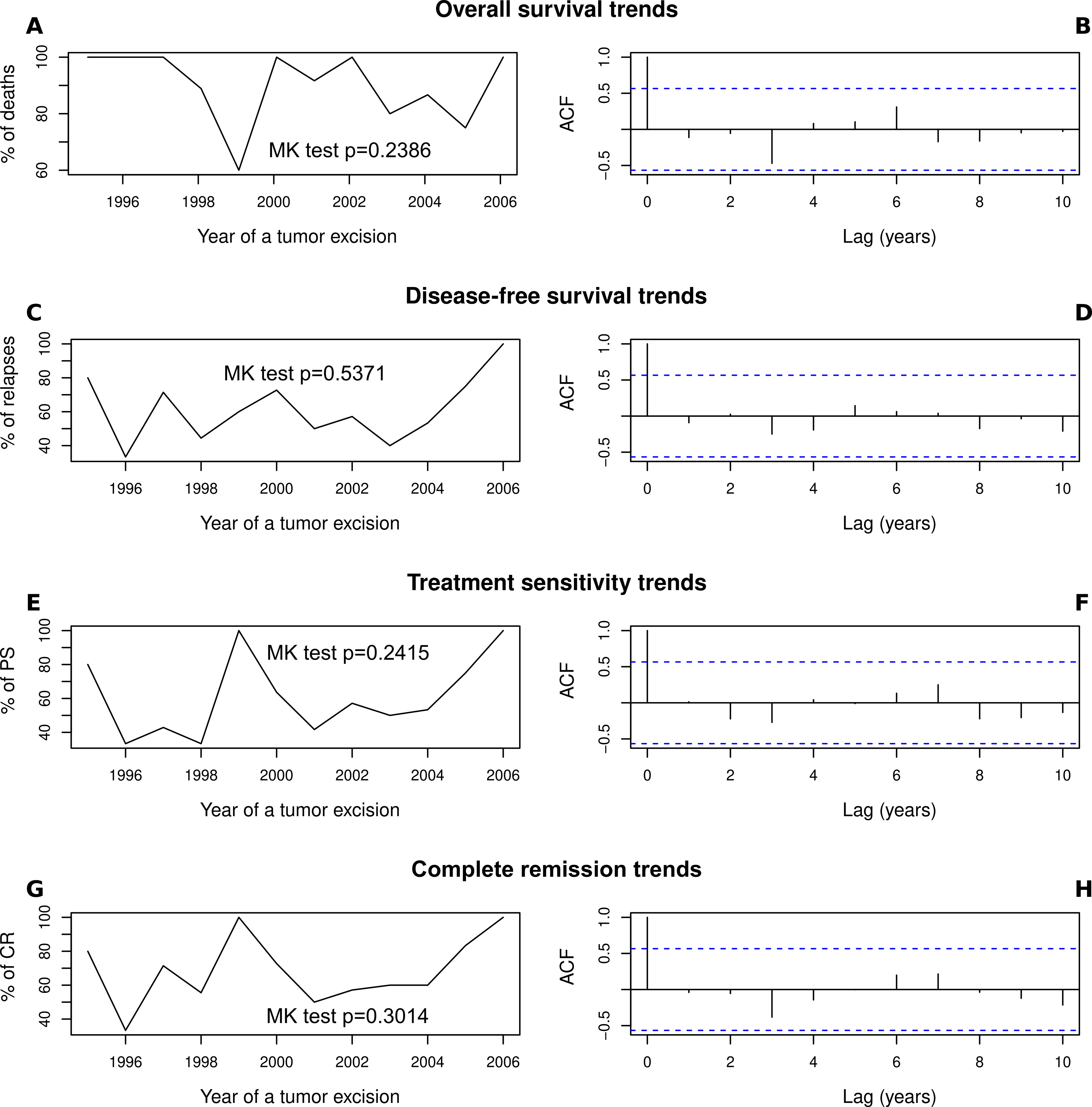

Supplement: S1 Fig — The patients underwent their first surgical treatment in the years 1995–2006. Time trends concerned overall survival (OS) (A,B); disease-free survival (DFS) (C,D); sensitivity to chemotherapy (PS) (E,F) and complete remission (CR) (G,H). The trends are shown as a trend line of death, relapse, PS and CR frequencies, respectively, supplemented with the results of the Mann-Kendall homogeneity test, and supported with autocorrelation function (ACF) plots. (TIF) [file pone.0271539.s001.tif]

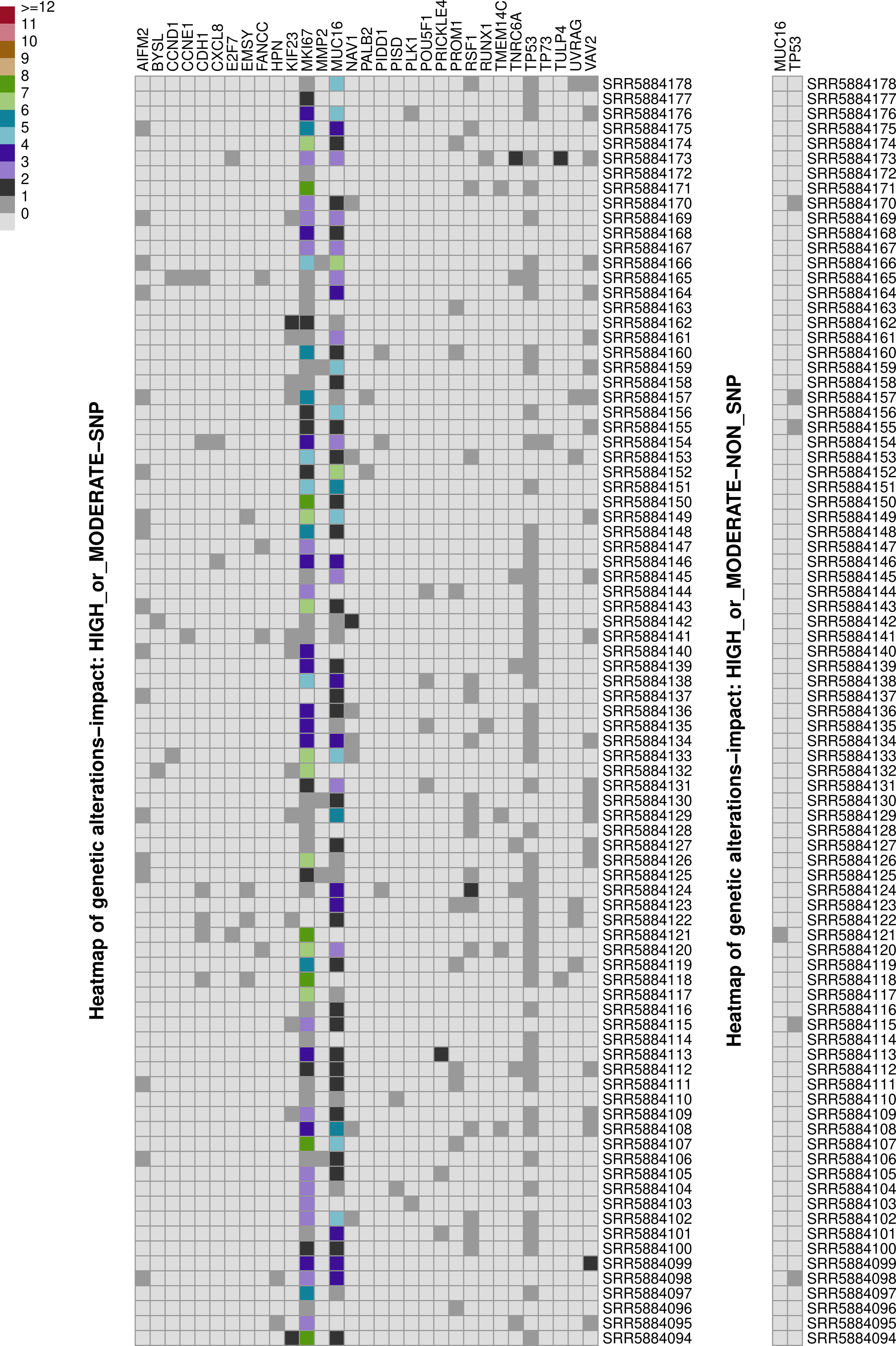

Supplement: S2 Fig — The results of the VEP analysis in the validation cohort of HGSOCs for 49 genes with significantly changed mRNA expression in the experimental cohort (only the genes with at least one sequence alteration in at least one sample are included). Abbreviations used: VEP–Variant Effect Prediction; HGSOCs–high-grade serous ovarian cancers. (TIF) [file pone.0271539.s002.tif]
