# Supplementary material for: PROM1, CXCL8, RUNX1, NAV1 and TP73 genes as independent markers predictive of prognosis or response to treatment in two cohorts of high-grade serous ovarian cancer patients
Source: PLoS One. 2022 Jul 22;17(7):e0271539. doi: 10.1371/journal.pone.0271539 (PMC9307210; doi:10.1371/journal.pone.0271539)
Supplement: S2 Table — (PDF) [file pone.0271539.s004.pdf]

Table S2. The significant results of the multivariate Cox and logistic regression analyses obtained for the experimental cohort of HGSOCs. The regularities subsequently confirmed in the validation cohort are emboldened and underlined.

	All samples					TP53 missense mutation: no					TP53 missense mutation: yes				
Analysis	HR/OR	95% CI	p-value	N	Ev. no.	HR/OR	95% CI	p-value	N	Ev. no.	HR/OR	95% CI	p-value	N	Ev. no.
**CR ~ CDKN1A**	0.3580	[0.136-0.941]	0.0372	69	50	-	-	-	-	-	-	-	-	-	-
RT >2 cm vs 0 cm	0.0668	[0.005-0.895]	0.0410	69	50	-	-	-	-	-	-	-	-	-	-
**CR ~ EMSY**	0.4879	[0.271-0.878]	0.0167	69	50	-	-	-	-	-	-	-	-	-	-
RT >2 cm vs 0 cm	0.0418	[0.002-0.983]	0.0488	69	50	-	-	-	-	-	-	-	-	-	-
**CR ~ NAV1**	**0.1461**	**[0.036-0.591]**	**0.0070**	**50**	**34**	-	-	-	-	-	<0.001	[0-0.462]	0.0278	29	21
**CR ~ STMN1**	-	-	-	-	-	<0.001	[0-0.514]	0.0347	27	19	-	-	-	-	-
**CR ~ UVRAG**	0.3967	[0.163-0.967]	0.0420	69	50	-	-	-	-	-	-	-	-	-	-
**PS ~ AIFM2**	0.4416	[0.215-0.907]	0.0260	69	45	<0.001	[0-0.53]	0.0337	27	16	-	-	-	-	-
RT >2 cm vs 0 cm	0.0583	[0.006-0.542]	0.0125	69	45	-	-	-	-	-	-	-	-	-	-
**PS ~ BBC3**	0.3849	[0.178-0.832]	0.0153	69	45	-	-	-	-	-	0.0877	[0.011-0.712]	0.0227	42	29
RT >2 cm vs 0 cm	0.0442	[0.004-0.46]	0.0091	69	45	-	-	-	-	-	0.0020	[0-0.522]	0.0287	42	29
**PS ~ CDK2**	0.4028	[0.187-0.868]	0.0202	69	45	-	-	-	-	-	0.2810	[0.085-0.924]	0.0367	42	29
RT <2 cm vs 0 cm	0.0882	[0.008-0.955]	0.0457	69	45	-	-	-	-	-	-	-	-	-	-
RT >2 cm vs 0 cm	0.0359	[0.003-0.472]	0.0114	69	45	-	-	-	-	-	0.0192	[0-0.92]	0.0452	42	29
**PS ~ CDK4**	0.4048	[0.183-0.894]	0.0253	69	45	-	-	-	-	-	0.2683	[0.087-0.832]	0.0227	42	29
RT <2 cm vs 0 cm	0.1118	[0.013-0.996]	0.0495	69	45	-	-	-	-	-	-	-	-	-	-
RT >2 cm vs 0 cm	0.0374	[0.003-0.432]	0.0085	69	45	-	-	-	-	-	0.0168	[0-0.726]	0.0335	42	29
**PS ~ CDKN1A**	0.3027	[0.112-0.819]	0.0186	69	45	-	-	-	-	-	0.1768	[0.034-0.923]	0.0399	42	29
RT >2 cm vs 0 cm	0.0378	[0.003-0.438]	0.0088	69	45	-	-	-	-	-	0.0165	[0-0.688]	0.0310	42	29
**PS ~ EMSY**	0.4250	[0.204-0.887]	0.0227	69	45	-	-	-	-	-	-	-	-	-	-
RT >2 cm vs 0 cm	0.0347	[0.003-0.441]	0.0095	69	45	-	-	-	-	-	-	-	-	-	-
**PS ~ FZD3**	0.1797	[0.052-0.627]	0.0071	50	29	-	-	-	-	-	-	-	-	-	-
RT >2 cm vs 0 cm	0.0305	[0.002-0.513]	0.0154	50	29	-	-	-	-	-	-	-	-	-	-
**PS ~ NAV1**	**0.2614**	**[0.08-0.851]**	**0.0259**	**50**	**29**	-	-	-	-	-	<0.001	[0-0.524]	0.0310	29	19
**PS ~ PALB2**	-	-	-	-	-	<0.001	[0-0.276]	0.0291	27	16	-	-	-	-	-
**PS ~ PDRG1**	0.3706	[0.162-0.847]	0.0186	69	45	<0.001	[0-0.164]	0.0249	27	16	-	-	-	-	-
RT >2 cm vs 0 cm	0.0468	[0.004-0.499]	0.0113	69	45	-	-	-	-	-	-	-	-	-	-
**PS ~ TNRC6A**	0.4293	[0.211-0.876]	0.0201	69	45	-	-	-	-	-	-	-	-	-	-
RT >2 cm vs 0 cm	0.0533	[0.005-0.572]	0.0155	69	45	-	-	-	-	-	-	-	-	-	-
**PS ~ TP73**	**0.5878**	**[0.347-0.996]**	**0.0482**	**69**	**45**	-	-	-	-	-	-	-	-	-	-
RT >2 cm vs 0 cm	**0.0861**	**[0.011-0.66]**	**0.0183**	**69**	**45**	-	-	-	-	-	-	-	-	-	-
**PS ~ UVRAG**	0.3936	[0.161-0.961]	0.0406	69	45	-	-	-	-	-	-	-	-	-	-
RT >2 cm vs 0 cm	0.0622	[0.006-0.628]	0.0185	69	45	-	-	-	-	-	-	-	-	-	-
**PS ~ ZBTB8A**	-	-	-	-	-	<0.001	[0-0.949]	0.0490	21	10	-	-	-	-	-
**DFS ~ AIFM2**	69.9047	[10.357-471.84]	0.0000	50	44	-	-	-	-	-	103.5758	[4.324->1000]	0.0042	31	27
FIGO IV vs IIB-IIC	14.5512	[1.217-173.985]	0.0344	50	44	-	-	-	-	-	25.6091	[1.541-425.48]	0.0237	31	27
RT <2 cm vs 0 cm	4.8623	[1.946-12.15]	0.0007	50	44	-	-	-	-	-	5.8012	[1.525-22.074]	0.0099	31	27
RT >2 cm vs 0 cm	2.9638	[1.013-8.674]	0.0474	50	44	-	-	-	-	-	-	-	-	-	-
**DFS ~ BBC3**	30.0562	[2.38-379.521]	0.0085	50	44	-	-	-	-	-	-	-	-	-	-
RT <2 cm vs 0 cm	5.0206	[1.918-13.14]	0.0010	50	44	-	-	-	-	-	-	-	-	-	-
RT >2 cm vs 0 cm	3.3106	[1.035-10.587]	0.0435	50	44	-	-	-	-	-	-	-	-	-	-
**DFS ~ BYSL**	-	-	-	-	-	>1000	[1.035->1000]	0.0490	19	17	-	-	-	-	-
RT <2 cm vs 0 cm	-	-	-	-	-	7.1284	[1.218-41.713]	0.0293	19	17	-	-	-	-	-
RT >2 cm vs 0 cm	-	-	-	-	-	17.8710	[1.928-165.606]	0.0111	19	17	-	-	-	-	-
**DFS ~ CCNE1**	-	-	-	-	-	935.2625	[1.389->1000]	0.0395	19	17	-	-	-	-	-
RT <2 cm vs 0 cm	-	-	-	-	-	10.7015	[1.689-67.774]	0.0118	19	17	-	-	-	-	-
RT >2 cm vs 0 cm	-	-	-	-	-	36.5619	[3.108-429.985]	0.0042	19	17	-	-	-	-	-
**DFS ~ CD44**	20.9970	[1.194-369.375]	0.0374	35	32	<0.001	[<0.001-0.261]	0.0385	13	13	283.0698	[5.55->1000]	0.0049	22	19
FIGO IV vs IIB-IIC	-	-	-	-	-	-	-	-	-	-	34.5188	[1.112->1000]	0.0433	22	19
RT <2 cm vs 0 cm	3.0909	[1.089-8.77]	0.0339	35	32	-	-	-	-	-	5.1171	[1.031-25.407]	0.0458	22	19
**DFS ~ CDK2**	8.7707	[1.202-63.996]	0.0322	50	44	207.8746	[1.270->1000]	0.0402	19	17	-	-	-	-	-
RT <2 cm vs 0 cm	4.0520	[1.631-10.069]	0.0026	50	44	10.3507	[1.774-60.365]	0.0094	19	17	-	-	-	-	-
RT >2 cm vs 0 cm	-	-	-	-	-	9.3836	[1.102-79.859]	0.0404	19	17	-	-	-	-	-
**DFS ~ CDK4**	14.7522	[1.732-125.674]	0.0138	50	44	-	-	-	-	-	-	-	-	-	-
RT <2 cm vs 0 cm	4.7547	[1.82-12.421]	0.0015	50	44	-	-	-	-	-	-	-	-	-	-
**DFS ~ CDKN1A**	>1000	[14.476->1000]	0.0067	50	44	-	-	-	-	-	-	-	-	-	-
FIGO IV vs IIB-IIC	12.8759	[1.128-147.01]	0.0397	50	44	-	-	-	-	-	-	-	-	-	-
RT <2 cm vs 0 cm	4.0445	[1.624-10.072]	0.0027	50	44	-	-	-	-	-	-	-	-	-	-
**DFS ~ CEBPA**	52.6598	[1.35->1000]	0.0340	34	31	-	-	-	-	-	-	-	-	-	-
**DFS ~ E2F7**	18.6653	[2.599-134.071]	0.0036	50	44	-	-	-	-	-	-	-	-	-	-
RT <2 cm vs 0 cm	4.5373	[1.778-11.577]	0.0016	50	44	-	-	-	-	-	-	-	-	-	-
**DFS ~ EMSY**	75.7903	[1.734->1000]	0.0247	50	44	>1000	[8.873->1000]	0.0193	19	17	-	-	-	-	-

	All samples					TP53 missense mutation: no					TP53 missense mutation: yes				
Analysis	HR/OR	95% CI	p-value	N	Ev. no.	HR/OR	95% CI	p-value	N	Ev. no.	HR/OR	95% CI	p-value	N	Ev. no.
RT <2 cm vs 0 cm	4.1972	[1.69-10.427]	0.0020	50	44	-	-	-	-	-	-	-	-	-	-
RT >2 cm vs 0 cm	-	-	-	-	-	20.9510	[2.361-185.895]	0.0063	19	17	-	-	-	-	-
**DFS ~ FZD3**	>1000	[181.59->1000]	0.0001	34	31	>1000	[63.244->1000]	0.0114	13	13	-	-	-	-	-
RT <2 cm vs 0 cm	4.6763	[1.571-13.917]	0.0056	34	31	-	-	-	-	-	-	-	-	-	-
RT >2 cm vs 0 cm	5.3583	[1.377-20.855]	0.0155	34	31	27.2086	[1.002-738.105]	0.0498	13	13	-	-	-	-	-
**DFS ~ GADD45A**	30.0838	[3.069-294.94]	0.0035	50	44	-	-	-	-	-	-	-	-	-	-
FIGO IV vs IIB-IIC	13.1922	[1.139-152.861]	0.0390	50	44	-	-	-	-	-	-	-	-	-	-
RT <2 cm vs 0 cm	3.9144	[1.61-9.517]	0.0026	50	44	-	-	-	-	-	-	-	-	-	-
**DFS ~ ING1**	-	-	-	-	-	>1000	[1.426->1000]	0.0389	13	13	-	-	-	-	-
**DFS ~ KIF23**	-	-	-	-	-	>1000	[24.047->1000]	0.0037	19	17	-	-	-	-	-
RT <2 cm vs 0 cm	-	-	-	-	-	13.5906	[1.936-95.374]	0.0087	19	17	-	-	-	-	-
RT >2 cm vs 0 cm	-	-	-	-	-	22.7086	[2.409-213.990]	0.0064	19	17	-	-	-	-	-
**DFS ~ MKI67**	-	-	-	-	-	<0.001	[<0.001-0.185]	0.0239	13	13	484.7565	[4.687->1000]	0.0090	22	19
RT <2 cm vs 0 cm	-	-	-	-	-	-	-	-	-	-	6.3294	[1.188-33.731]	0.0307	22	19
**DFS ~ MMP2**	>1000	[18.116->1000]	0.0043	35	32	-	-	-	-	-	>1000	[>1000->1000]	0.0005	22	19
FIGO IIIA-IIIB vs IIB-IIC	-	-	-	-	-	-	-	-	-	-	101.7101	[3.026->1000]	0.0100	22	19
FIGO IV vs IIB-IIC	-	-	-	-	-	-	-	-	-	-	972.7769	[12.127->1000]	0.0021	22	19
RT <2 cm vs 0 cm	2.9581	[1.059-8.26]	0.0384	35	32	-	-	-	-	-	-	-	-	-	-
**DFS ~ MUC16**	-	-	-	-	-	0.0128	[0.000-0.909]	0.0451	13	13	-	-	-	-	-
**DFS ~ PDRG1**	14.5843	[2.315-91.889]	0.0043	50	44	144.1748	[1.130->1000]	0.0445	19	17	-	-	-	-	-
FIGO IV vs IIB-IIC	13.0229	[1.15-147.462]	0.0382	50	44	-	-	-	-	-	-	-	-	-	-
RT <2 cm vs 0 cm	3.3605	[1.391-8.12]	0.0071	50	44	-	-	-	-	-	-	-	-	-	-
RT >2 cm vs 0 cm	-	-	-	-	-	9.9637	[1.145-86.635]	0.0372	19	17	-	-	-	-	-
**DFS ~ PIDD1**	-	-	-	-	-	>1000	[1.432->1000]	0.0415	19	17	-	-	-	-	-
**DFS ~ PISD**	5.7026	[1.074-30.273]	0.0410	50	44	-	-	-	-	-	-	-	-	-	-
RT <2 cm vs 0 cm	3.8090	[1.527-9.502]	0.0041	50	44	-	-	-	-	-	-	-	-	-	-
**DFS ~ PLK1**	-	-	-	-	-	>1000	[2.380->1000]	0.0294	19	17	-	-	-	-	-
RT <2 cm vs 0 cm	-	-	-	-	-	9.3272	[1.531-56.823]	0.0154	19	17	-	-	-	-	-
RT >2 cm vs 0 cm	-	-	-	-	-	15.0380	[1.863-121.369]	0.0110	19	17	-	-	-	-	-
**DFS ~ POU5F1**	-	-	-	-	-	-	-	-	-	-	>1000	[12.64->1000]	0.0041	22	19
FIGO IIIA-IIIB vs IIB-IIC	-	-	-	-	-	-	-	-	-	-	26.0523	[1.026-661.522]	0.0482	22	19
FIGO IV vs IIB-IIC	-	-	-	-	-	-	-	-	-	-	210.9877	[4.076->1000]	0.0079	22	19
**DFS ~ PRICKLE4**	46.1786	[3.545-601.585]	0.0034	50	44	758.5218	[2.661->1000]	0.0215	19	17	-	-	-	-	-
FIGO IV vs IIB-IIC	14.0475	[1.231-160.25]	0.0334	50	44	-	-	-	-	-	-	-	-	-	-
RT <2 cm vs 0 cm	4.6520	[1.863-11.614]	0.0010	50	44	-	-	-	-	-	-	-	-	-	-
**DFS ~ PROM1**	59.1797	[3.66-956.967]	0.0041	35	32	-	-	-	-	-	**>1000**	**[45.567->1000]**	**0.0006**	**22**	**19**
FIGO IIIA-IIIB vs IIB-IIC	19.0412	[1.335-271.549]	0.0298	35	32	-	-	-	-	-	**221.4353**	**[4.088->1000]**	**0.0080**	**22**	**19**
FIGO IIIC vs IIB-IIC	-	-	-	-	-	-	-	-	-	-	**108.8040**	**[2.608->1000]**	**0.0138**	**22**	**19**
FIGO IV vs IIB-IIC	28.6215	[1.711-478.705]	0.0196	35	32	-	-	-	-	-	**>1000**	**[12.001->1000]**	**0.0033**	**22**	**19**
RT <2 cm vs 0 cm	2.7335	[1.001-7.462]	0.0497	35	32	-	-	-	-	-	-	-	-	-	-
**DFS ~ RSF1**	164.1895	[1.878->1000]	0.0253	50	44	>1000	[32.549->1000]	0.0147	19	17	-	-	-	-	-
RT <2 cm vs 0 cm	4.0405	[1.624-10.053]	0.0027	50	44	-	-	-	-	-	-	-	-	-	-
RT >2 cm vs 0 cm	-	-	-	-	-	23.0302	[2.302-230.397]	0.0076	19	17	-	-	-	-	-
**DFS ~ RUNX1**	-	-	-	-	-	**<0.001**	**[<0.001-0.395]**	**0.0257**	**13**	**13**	-	-	-	-	-
**DFS ~ RUNX2**	-	-	-	-	-	<0.001	[<0.001-0.288]	0.0313	13	13	>1000	[38.657->1000]	0.0159	22	19
FIGO IIIA-IIIB vs IIB-IIC	-	-	-	-	-	-	-	-	-	-	569.4710	[3.926->1000]	0.0125	22	19
FIGO IIIC vs IIB-IIC	-	-	-	-	-	-	-	-	-	-	199.4673	[2.43->1000]	0.0185	22	19
FIGO IV vs IIB-IIC	-	-	-	-	-	-	-	-	-	-	>1000	[11.118->1000]	0.0036	22	19
**DFS ~ SNRPD3**	109.8511	[4.063->1000]	0.0052	50	44	-	-	-	-	-	659.8314	[2.511->1000]	0.0224	31	27
FIGO IV vs IIB-IIC	-	-	-	-	-	-	-	-	-	-	30.5868	[1.791-522.329]	0.0182	31	27
RT <2 cm vs 0 cm	3.3317	[1.388-8]	0.0071	50	44	-	-	-	-	-	-	-	-	-	-
**DFS ~ TCEA3**	21.3068	[2.41-188.406]	0.0059	50	44	-	-	-	-	-	-	-	-	-	-
RT <2 cm vs 0 cm	3.2835	[1.353-7.97]	0.0086	50	44	-	-	-	-	-	-	-	-	-	-
**DFS ~ TMEM14C**	12.9709	[1.764-95.388]	0.0118	50	44	>1000	[48.007->1000]	0.0030	19	17	-	-	-	-	-
FIGO IIIC vs IIB-IIC	-	-	-	-	-	0.0355	[0.001-0.768]	0.0333	19	17	-	-	-	-	-
RT <2 cm vs 0 cm	3.8919	[1.592-9.517]	0.0029	50	44	8.9319	[1.806-44.161]	0.0072	19	17	-	-	-	-	-
RT >2 cm vs 0 cm	-	-	-	-	-	38.6844	[3.999-374.185]	0.0016	19	17	-	-	-	-	-
**DFS ~ TNRC6A**	5.1758	[1.1-24.356]	0.0375	50	44	-	-	-	-	-	-	-	-	-	-
RT <2 cm vs 0 cm	4.2527	[1.645-10.993]	0.0028	50	44	-	-	-	-	-	-	-	-	-	-
**DFS ~ TP53**	-	-	-	-	-	62.0322	[1.073->1000]	0.0461	19	17	-	-	-	-	-
RT <2 cm vs 0 cm	-	-	-	-	-	9.3817	[1.444-60.933]	0.0190	19	17	-	-	-	-	-
RT >2 cm vs 0 cm	-	-	-	-	-	11.1259	[1.403-88.226]	0.0226	19	17	-	-	-	-	-
**DFS ~ TP53INP1**	51.8442	[4.025-667.806]	0.0025	50	44	-	-	-	-	-	188.9710	[3.104->1000]	0.0124	31	27
FIGO IV vs IIB-IIC	17.6125	[1.425-217.664]	0.0253	50	44	-	-	-	-	-	57.1601	[3.075->1000]	0.0067	31	27
RT <2 cm vs 0 cm	2.7261	[1.129-6.585]	0.0258	50	44	-	-	-	-	-	-	-	-	-	-

	All samples					TP53 missense mutation: no					TP53 missense mutation: yes				
Analysis	HR/OR	95% CI	p-value	N	Ev. no.	HR/OR	95% CI	p-value	N	Ev. no.	HR/OR	95% CI	p-value	N	Ev. no.
**DFS ~ UVRAG**	29.0148	[1.24-678.916]	0.0363	50	44	-	-	-	-	-	-	-	-	-	-
FIGO IV vs IIB-IIC	11.5497	[1.026-129.982]	0.0476	50	44	-	-	-	-	-	-	-	-	-	-
RT <2 cm vs 0 cm	3.5205	[1.459-8.495]	0.0051	50	44	-	-	-	-	-	-	-	-	-	-
**DFS ~ ZBTB8A**	61.4272	[1.967->1000]	0.0190	34	31	>1000	[2.471->1000]	0.0269	13	13	-	-	-	-	-
**OS ~ AIFM2**	19.3951	[4.791-78.513]	0.0000	69	60	27.2976	[1.986-375.200]	0.0134	27	25	25.9394	[2.872-234.245]	0.0037	42	35
RT <2 cm vs 0 cm	4.5056	[1.965-10.33]	0.0004	69	60	-	-	-	-	-	5.3506	[1.653-17.317]	0.0051	42	35
RT >2 cm vs 0 cm	4.5399	[1.758-11.727]	0.0018	69	60	8.9465	[1.582-50.563]	0.0131	27	25	3.9166	[1.179-13.012]	0.0258	42	35
**OS ~ BBC3**	12.6436	[1.95-81.977]	0.0078	69	60	-	-	-	-	-	-	-	-	-	-
RT <2 cm vs 0 cm	4.9695	[2.048-12.058]	0.0004	69	60	-	-	-	-	-	-	-	-	-	-
RT >2 cm vs 0 cm	5.2087	[1.942-13.97]	0.0010	69	60	-	-	-	-	-	-	-	-	-	-
**OS ~ CCND1**	-	-	-	-	-	245.8935	[7.423->1000]	0.0021	27	25	-	-	-	-	-
RT >2 cm vs 0 cm	-	-	-	-	-	22.9554	[3.069-171.699]	0.0023	27	25	-	-	-	-	-
**OS ~ CDH1**	11.3467	[1.2-107.298]	0.0341	51	45	-	-	-	-	-	-	-	-	-	-
RT <2 cm vs 0 cm	3.2428	[1.275-8.247]	0.0135	51	45	-	-	-	-	-	-	-	-	-	-
RT >2 cm vs 0 cm	3.4121	[1.135-10.256]	0.0288	51	45	-	-	-	-	-	-	-	-	-	-
**OS ~ CDK2**	5.8860	[1.41-24.573]	0.0151	69	60	-	-	-	-	-	-	-	-	-	-
RT <2 cm vs 0 cm	4.7537	[1.927-11.729]	0.0007	69	60	-	-	-	-	-	-	-	-	-	-
RT >2 cm vs 0 cm	4.9910	[1.823-13.664]	0.0018	69	60	-	-	-	-	-	-	-	-	-	-
**OS ~ CDK4**	6.9917	[1.152-42.44]	0.0345	69	60	-	-	-	-	-	-	-	-	-	-
RT <2 cm vs 0 cm	4.5941	[1.88-11.229]	0.0008	69	60	-	-	-	-	-	-	-	-	-	-
RT >2 cm vs 0 cm	5.5088	[1.964-15.449]	0.0012	69	60	-	-	-	-	-	-	-	-	-	-
**OS ~ CDKN1A**	>1000	[27.506->1000]	0.0013	69	60	-	-	-	-	-	>1000	[5.903->1000]	0.0137	42	35
RT <2 cm vs 0 cm	4.6296	[1.932-11.096]	0.0006	69	60	-	-	-	-	-	5.3056	[1.596-17.636]	0.0065	42	35
RT >2 cm vs 0 cm	5.6532	[2.069-15.448]	0.0007	69	60	-	-	-	-	-	5.2010	[1.402-19.294]	0.0137	42	35
**OS ~ CXCL8**	-	-	-	-	-	**34.5639**	**[1.560-765.542]**	**0.0250**	**21**	**21**	-	-	-	-	-
**OS ~ DNMT3A**	9.4132	[1.042-85.058]	0.0459	51	45	-	-	-	-	-	-	-	-	-	-
RT <2 cm vs 0 cm	3.1975	[1.249-8.185]	0.0154	51	45	-	-	-	-	-	-	-	-	-	-
RT >2 cm vs 0 cm	3.7324	[1.25-11.142]	0.0183	51	45	-	-	-	-	-	-	-	-	-	-
**OS ~ EMSY**	14.7737	[2.827-77.21]	0.0014	69	60	95.9945	[3.746->1000]	0.0058	27	25	15.2888	[1.464-159.674]	0.0227	42	35
RT <2 cm vs 0 cm	4.4816	[1.892-10.616]	0.0007	69	60	-	-	-	-	-	4.7098	[1.488-14.908]	0.0084	42	35
RT >2 cm vs 0 cm	5.3148	[1.969-14.342]	0.0010	69	60	14.2789	[2.077-98.125]	0.0069	27	25	4.4319	[1.278-15.364]	0.0189	42	35
**OS ~ FANCC**	12.4719	[1.345-115.626]	0.0264	69	60	-	-	-	-	-	-	-	-	-	-
RT <2 cm vs 0 cm	4.7174	[1.94-11.47]	0.0006	69	60	-	-	-	-	-	-	-	-	-	-
RT >2 cm vs 0 cm	5.4782	[1.997-15.027]	0.0010	69	60	-	-	-	-	-	-	-	-	-	-
**OS ~ FZD3**	35.6165	[1.085->1000]	0.0449	50	44	-	-	-	-	-	-	-	-	-	-
RT <2 cm vs 0 cm	4.3859	[1.557-12.351]	0.0051	50	44	-	-	-	-	-	-	-	-	-	-
RT >2 cm vs 0 cm	6.5622	[1.97-21.857]	0.0022	50	44	-	-	-	-	-	-	-	-	-	-
**OS ~ GADD45A**	5.0790	[1.001-25.759]	0.0498	69	60	-	-	-	-	-	-	-	-	-	-
RT <2 cm vs 0 cm	3.9992	[1.708-9.366]	0.0014	69	60	-	-	-	-	-	-	-	-	-	-
RT >2 cm vs 0 cm	4.4119	[1.664-11.698]	0.0029	69	60	-	-	-	-	-	-	-	-	-	-
**OS ~ HPN**	5.1655	[1.029-25.94]	0.0461	50	44	-	-	-	-	-	-	-	-	-	-
RT <2 cm vs 0 cm	3.5806	[1.316-9.743]	0.0125	50	44	-	-	-	-	-	-	-	-	-	-
RT >2 cm vs 0 cm	4.8243	[1.552-15]	0.0065	50	44	-	-	-	-	-	-	-	-	-	-
**OS ~ KIF23**	7.2506	[1.562-33.661]	0.0114	69	60	63.3377	[2.417->1000]	0.0128	27	25	-	-	-	-	-
RT <2 cm vs 0 cm	4.8695	[1.999-11.861]	0.0005	69	60	5.0200	[1.120-22.498]	0.0350	27	25	-	-	-	-	-
RT >2 cm vs 0 cm	4.6164	[1.769-12.045]	0.0018	69	60	8.9781	[1.521-52.990]	0.0154	27	25	-	-	-	-	-
**OS ~ MKI67**	-	-	-	-	-	<0.001	[<0.001-0.008]	0.0022	21	21	-	-	-	-	-
FIGO IIIA-IIIB vs IIB-IIC	-	-	-	-	-	13.4236	[1.334-135.012]	0.0274	21	21	-	-	-	-	-
**OS ~ MUC16**	-	-	-	-	-	0.0217	[0.001-0.440]	0.0127	21	21	-	-	-	-	-
FIGO IIIA-IIIB vs IIB-IIC	-	-	-	-	-	12.9894	[1.238-136.225]	0.0325	21	21	-	-	-	-	-
**OS ~ NAV1**	-	-	-	-	-	-	-	-	-	-	>1000	[1.971->1000]	0.0371	29	23
RT <2 cm vs 0 cm	-	-	-	-	-	-	-	-	-	-	15.7298	[1.814-136.362]	0.0124	29	23
RT >2 cm vs 0 cm	-	-	-	-	-	-	-	-	-	-	28.4444	[2.868-282.08]	0.0042	29	23
**OS ~ PDRG1**	14.8770	[3.32-66.666]	0.0004	69	60	-	-	-	-	-	-	-	-	-	-
RT <2 cm vs 0 cm	4.4792	[1.906-10.528]	0.0006	69	60	-	-	-	-	-	-	-	-	-	-
RT >2 cm vs 0 cm	5.5545	[2.052-15.033]	0.0007	69	60	-	-	-	-	-	-	-	-	-	-
**OS ~ PIDD1**	6.8403	[1.387-33.74]	0.0182	69	60	551.0993	[1.295->1000]	0.0410	27	25	-	-	-	-	-
RT <2 cm vs 0 cm	5.3756	[2.04-14.167]	0.0007	69	60	-	-	-	-	-	-	-	-	-	-
RT >2 cm vs 0 cm	6.2576	[2.117-18.498]	0.0009	69	60	7.4679	[1.177-47.355]	0.0329	27	25	-	-	-	-	-
**OS ~ PISD**	6.8386	[1.353-34.571]	0.0200	69	60	-	-	-	-	-	12.3203	[1.108-136.981]	0.0410	42	35
RT <2 cm vs 0 cm	4.1825	[1.802-9.706]	0.0009	69	60	-	-	-	-	-	6.1343	[1.567-24.008]	0.0092	42	35
RT >2 cm vs 0 cm	5.4955	[2.02-14.949]	0.0008	69	60	-	-	-	-	-	6.0898	[1.41-26.305]	0.0155	42	35
**OS ~ PROM1**	-	-	-	-	-	9.2416	[1.274-67.005]	0.0278	21	21	-	-	-	-	-
**OS ~ RSF1**	4.8531	[1.155-20.396]	0.0311	69	60	376.6904	[4.970->1000]	0.0072	27	25	-	-	-	-	-
RT <2 cm vs 0 cm	3.9307	[1.675-9.223]	0.0017	69	60	-	-	-	-	-	-	-	-	-	-

	All samples					TP53 missense mutation: no					TP53 missense mutation: yes				
Analysis	HR/OR	95% CI	p-value	N	Ev. no.	HR/OR	95% CI	p-value	N	Ev. no.	HR/OR	95% CI	p-value	N	Ev. no.
RT >2 cm vs 0 cm	4.7191	[1.769-12.592]	0.0019	69	60	13.6723	[1.979-94.412]	0.0080	27	25	-	-	-	-	-
**OS ~ SFN**	-	-	-	-	-	>1000	[296.775->1000]	0.0222	21	21	-	-	-	-	-
**OS ~ SNRPD3**	54.9429	[2.551->1000]	0.0105	69	60	-	-	-	-	-	183.9518	[2.696->1000]	0.0155	42	35
RT <2 cm vs 0 cm	4.1716	[1.813-9.597]	0.0008	69	60	-	-	-	-	-	4.9464	[1.507-16.24]	0.0084	42	35
RT >2 cm vs 0 cm	5.2580	[1.983-13.94]	0.0008	69	60	-	-	-	-	-	4.9062	[1.337-18.003]	0.0165	42	35
**OS ~ TNRC6A**	5.0773	[1.287-20.036]	0.0204	69	60	-	-	-	-	-	-	-	-	-	-
RT <2 cm vs 0 cm	4.2786	[1.852-9.886]	0.0007	69	60	-	-	-	-	-	-	-	-	-	-
RT >2 cm vs 0 cm	4.7143	[1.812-12.268]	0.0015	69	60	-	-	-	-	-	-	-	-	-	-
**OS ~ TP53**	-	-	-	-	-	49.8353	[1.364->1000]	0.0332	27	25	-	-	-	-	-
RT <2 cm vs 0 cm	-	-	-	-	-	7.1061	[1.332-37.900]	0.0217	27	25	-	-	-	-	-
RT >2 cm vs 0 cm	-	-	-	-	-	16.6969	[2.368-117.709]	0.0047	27	25	-	-	-	-	-
**OS ~ TP53INP1**	9.0175	[1.436-56.64]	0.0190	69	60	-	-	-	-	-	16.7214	[1.469-190.285]	0.0232	42	35
RT <2 cm vs 0 cm	3.3352	[1.444-7.702]	0.0048	69	60	-	-	-	-	-	3.6216	[1.172-11.195]	0.0254	42	35
RT >2 cm vs 0 cm	3.2664	[1.211-8.813]	0.0194	69	60	-	-	-	-	-	-	-	-	-	-
**OS ~ TULP4**	-	-	-	-	-	31.5796	[1.279-779.133]	0.0348	21	21	-	-	-	-	-
**OS ~ UVRAG**	11.8654	[1.924-73.192]	0.0077	69	60	32.7140	[1.528-700.025]	0.0256	27	25	-	-	-	-	-
RT <2 cm vs 0 cm	4.0234	[1.73-9.356]	0.0012	69	60	-	-	-	-	-	-	-	-	-	-
RT >2 cm vs 0 cm	4.8320	[1.821-12.823]	0.0016	69	60	8.6148	[1.414-52.468]	0.0195	27	25	-	-	-	-	-
**OS ~ VAV2**	17.9321	[2.103-152.913]	0.0083	50	44	>1000	[33.027->1000]	0.0008	21	21	-	-	-	-	-
RT >2 cm vs 0 cm	5.3510	[1.73-16.555]	0.0036	50	44	-	-	-	-	-	-	-	-	-	-

Abbreviations used: CR – Complete Remission; DFS – Disease-Free Survival, FIGO – clinical stage; OS – Overall Survival; PS – Platinum Sensitivity; RT – Residual Tumor; HGSOC – high-grade serous ovarian cancer; HR – Hazard Ratio; OR – Odds Ratio; CI – Confidence Interval; Ev. no. – number of events (deaths, recurrences, PSs, CRs)
