# Supplementary material for: PROM1, CXCL8, RUNX1, NAV1 and TP73 genes as independent markers predictive of prognosis or response to treatment in two cohorts of high-grade serous ovarian cancer patients
Source: PLoS One. 2022 Jul 22;17(7):e0271539. doi: 10.1371/journal.pone.0271539 (PMC9307210; doi:10.1371/journal.pone.0271539)
Supplement: S4 Table — (PDF) [file pone.0271539.s006.pdf]

Table S4. Summary of the VEP analysis in the validation cohort for 49 genes differentially expressed in the experimental cohort.

Gene	Significant	Validated	SNP variants (high)	SNP variants (high/ mod)	New unique SNP variants (high)	New unique SNP variants (high/ mod)	NON-SNP variants (high)	NON-SNP variants (high/ mod)	New unique NON-SNP variants (high)	New unique NON-SNP variants (high/ mod)	Altered samples (high)	Freq. of altered samples (high)	Altered samples (high/ mod)	Freq. of altered samples (high/ mod)
**CXCL8**	Yes	Yes	2	2	0	0	0	0	0	0	2	0.024	2	0.024
**NAV1**	Yes	Yes	1	9	1	3	0	0	0	0	1	0.012	8	0.094
**PROM1**	Yes	Yes	9	10	1	1	0	0	0	0	9	0.106	10	0.118
**RUNX1**	Yes	Yes	1	2	0	0	0	0	0	0	1	0.012	2	0.024
**TP73**	Yes	Yes	0	1	0	0	0	0	0	0	0	0.000	1	0.012
**AIFM2**	Yes	No	0	17	0	0	0	0	0	0	0	0.000	17	0.200
**BBC3**	Yes	No	0	0	0	0	0	0	0	0	0	0.000	0	0.000
**BYSL**	Yes	No	0	2	0	0	0	0	0	0	0	0.000	2	0.024
**CCND1**	Yes	No	0	2	0	1	0	0	0	0	0	0.000	2	0.024
**CCNE1**	Yes	No	0	2	0	0	0	0	0	0	0	0.000	2	0.024
**CD44**	Yes	No	0	0	0	0	0	0	0	0	0	0.000	0	0.000
**CDH1**	Yes	No	1	6	1	1	0	0	0	0	1	0.012	6	0.071
**CDK2**	Yes	No	0	0	0	0	0	0	0	0	0	0.000	0	0.000
**CDK4**	Yes	No	0	0	0	0	0	0	0	0	0	0.000	0	0.000
**CDKN1A**	Yes	No	0	0	0	0	0	0	0	0	0	0.000	0	0.000
**CEBPA**	Yes	No	0	0	0	0	0	0	0	0	0	0.000	0	0.000
**DNMT3A**	Yes	No	0	0	0	0	0	0	0	0	0	0.000	0	0.000
**E2F7**	Yes	No	0	2	0	0	0	0	0	0	0	0.000	2	0.024
**EMSY**	Yes	No	0	4	0	0	0	0	0	0	0	0.000	4	0.047
**FANCC**	Yes	No	0	4	0	1	0	0	0	0	0	0.000	4	0.047
**FZD3**	Yes	No	0	0	0	0	0	0	0	0	0	0.000	0	0.000
**GADD45A**	Yes	No	0	0	0	0	0	0	0	0	0	0.000	0	0.000
**HPN**	Yes	No	0	2	0	0	0	0	0	0	0	0.000	2	0.024
**ING1**	Yes	No	0	0	0	0	0	0	0	0	0	0.000	0	0.000
**KIF23**	Yes	No	1	15	1	3	0	0	0	0	1	0.012	13	0.153
**MKI67**	Yes	No	0	291	0	1	0	0	0	0	0	0.000	82	0.965
**MMP2**	Yes	No	4	5	0	0	0	0	0	0	4	0.047	5	0.059
**MUC16**	Yes	No	123	204	4	8	1	1	0	0	58	0.682	71	0.835
**PALB2**	Yes	No	0	2	0	0	0	0	0	0	0	0.000	2	0.024
**PDRG1**	Yes	No	0	0	0	0	0	0	0	0	0	0.000	0	0.000
**PIDD1**	Yes	No	0	3	0	0	0	0	0	0	0	0.000	3	0.035
**PISD**	Yes	No	0	2	0	0	0	0	0	0	0	0.000	2	0.024
**PLK1**	Yes	No	0	2	0	1	0	0	0	0	0	0.000	2	0.024
**POU5F1**	Yes	No	0	4	0	0	0	0	0	0	0	0.000	4	0.047
**PRICKLE4**	Yes	No	0	4	0	0	0	0	0	0	0	0.000	3	0.035
**RSF1**	Yes	No	12	19	1	3	0	0	0	0	12	0.141	18	0.212
**RUNX2**	Yes	No	0	0	0	0	0	0	0	0	0	0.000	0	0.000
**SFN**	Yes	No	0	0	0	0	0	0	0	0	0	0.000	0	0.000
**SNRPD3**	Yes	No	0	0	0	0	0	0	0	0	0	0.000	0	0.000
**STMN1**	Yes	No	0	0	0	0	0	0	0	0	0	0.000	0	0.000
**TCEA3**	Yes	No	0	0	0	0	0	0	0	0	0	0.000	0	0.000
**TMEM14C**	Yes	No	4	4	1	1	0	0	0	0	4	0.047	4	0.047
**TNRC6A**	Yes	No	0	9	0	1	0	0	0	0	0	0.000	8	0.094
**TP53**	Yes	No	6	54	0	0	5	5	2	2	11	0.129	59	0.694
**TP53INP1**	Yes	No	0	0	0	0	0	0	0	0	0	0.000	0	0.000
**TULP4**	Yes	No	0	3	0	0	0	0	0	0	0	0.000	2	0.024
**UVRAG**	Yes	No	1	6	1	2	0	0	0	0	1	0.012	6	0.071
**VAV2**	Yes	No	21	24	1	1	0	0	0	0	21	0.247	23	0.271
**ZBTB8A**	Yes	No	0	0	0	0	0	0	0	0	0	0.000	0	0.000
		**Sum:**	186	716	12	28	6	6	2	2	NA	NA	NA	NA

Green color denotes the results for the genes the expression of which was successfully validated in an independent cohort of 85 HGSOC samples. If more than 90% of samples had the number of normalized NGS RNA-seq reads equaling 0 for the specified gene, the results are shown in red. Abbreviations used: VEP – Variant Effect Prediction; HGSOC – high-grade serous ovarian cancer
